# Using THz Spectroscopy, Evolutionary Network Analysis Methods, and MD Simulation to Map the Evolution of Allosteric Communication Pathways in c-Type Lysozymes

**DOI:** 10.1093/molbev/msv178

**Published:** 2015-09-03

**Authors:** Kristina N. Woods, Juergen Pfeffer

**Affiliations:** ^1^Physics Department, Carnegie Mellon University; ^2^Institute for Software Research, Carnegie Mellon University

**Keywords:** THz spectroscopy, protein dynamics, sequence evolution

## Abstract

It is now widely accepted that protein function is intimately tied with the navigation of energy landscapes. In this framework, a protein sequence is not described by a distinct structure but rather by an ensemble of conformations. And it is through this ensemble that evolution is able to modify a protein’s function by altering its landscape. Hence, the evolution of protein functions involves selective pressures that adjust the sampling of the conformational states. In this work, we focus on elucidating the evolutionary pathway that shaped the function of individual proteins that make-up the mammalian c-type lysozyme subfamily. Using both experimental and computational methods, we map out specific intermolecular interactions that direct the sampling of conformational states and accordingly, also underlie shifts in the landscape that are directly connected with the formation of novel protein functions. By contrasting three representative proteins in the family we identify molecular mechanisms that are associated with the selectivity of enhanced antimicrobial properties and consequently, divergent protein function. Namely, we link the extent of localized fluctuations involving the loop separating helices A and B with shifts in the equilibrium of the ensemble of conformational states that mediate interdomain coupling and concurrently moderate substrate binding affinity. This work reveals unique insights into the molecular level mechanisms that promote the progression of interactions that connect the immune response to infection with the nutritional properties of lactation, while also providing a deeper understanding about how evolving energy landscapes may define present-day protein function.

## Introduction

The origin of long-range communication in proteins has been attributed to dynamic interactions of amino acids in the protein three-dimensional (3D) structure (Süel et al. 2003; del Sol et al. 2006; Hilser and Thompson 2007; Reynolds et al. 2011; Nussinov 2012). Incidentally, this long-distance communication and hence, intraprotein signaling has been conjectured to play an important role in determining overall protein function. For instance, external perturbations such as ligand-binding have been found to prompt a long-distance response in many proteins (Niessen et al. 2014; Gagné et al. 2015). The local perturbation from binding frequently extends to remote sites in the protein tertiary structure that are manifested as fluctuations in the positions or mobility of amino acid residues that alter the dynamical landscape of the protein. By initiating changes in the surrounding environment, ligand-binding shifts the energy landscape of the protein and promotes redistribution within the already preexisting population of protein conformational states (Betts and Sternberg 1999; Goh et al. 2004) (supplementary material S1, fig. S1, Supplementary Material online). The underlying origin of the long-range signal propagation mechanism or allostery has been ascribed to excitation of protein global fluctuations (Okazaki and Takada 2008; Bahar 2010; Meireles et al. 2011; Nussinov and Tsai 2013). These global motions are thought to be encoded by the evolutionarily conserved residues within the protein family (Bahar et al. 2007; Liu and Bahar 2012) and are ultimately connected energetically by large-scale concerted protein fluctuations. In general, allostery in proteins enables the activity of one site in a protein to modulate function at another spatially distinct region. Although interestingly, it is only a small subset of the protein residues that are believed to participate in forming physically coupled interactions that link distant functional sites in the 3D protein structure that ultimately constitute the global fluctuations (Süel et al. 2003; Reynolds et al. 2011). In fact, recent developments in both experimental and computational modeling of allosteric regulation in protein systems have indicated that internal motions on the subnanosecond time scale are important facilitating the large-scale collective global motions in proteins (Frederick et al. 2007; Motlagh et al. 2014). These fast, internal protein fluctuations initiate allosteric conformational changes by altering the dynamic ensemble of protein conformational substates (Kumar et al. 2000; Ishikawa et al. 2008). In other words, perturbations at specific sites within proteins may cause conformational changes to take place through a diverse set of interactions that are not necessarily apparent from examining the structure of the protein alone. Further, it has been conjectured that through these composite interactions that signal flow is mediated and mechanisms such as allosteric regulation and specificity of molecular recognition are born (Li et al. 2014). The evolutionarily conserved “sparse” network of amino acid interactions represents a structural motif for allosteric communication in proteins (Halabi et al. 2009; Reynolds et al. 2011). So from an evolutionary perspective, sequences that adopt a conformational ensemble can transmit biological divergence through selective pressures (Süel et al. 2003; Wagner 2003; Meier and Ozbek 2007; Halabi et al. 2009). Sequence changes alter the equilibrium of the conformational ensembles and in effect may also cause a loss or a gain of a subset of functions. Therefore, on evolutionary time scales, changes in the equilibrium of a conformational ensemble may result in a highly diverse collection of conformational states among structurally similar proteins.

In this study, we focus our efforts on experimental and computational methods that are capable of mapping globally energetic interactions between amino acid residues that may be able to provide insight into the evolutionary networks and consequently, progression of function of members of proteins in a single family. Specifically, we use terahertz (THz) time-scale infrared spectroscopy combined with molecular dynamics (MD) simulation and sequence alignment profiles to probe the long-range dynamical and conformational effects of ligand-binding of proteins in the c-type lysozyme subfamily. Our experimental aim is to determine how the detected fast (picosecond time-scale), intraprotein dynamics are tied with the formation of allosteric propagation pathways (Woods 2014c) that link distant interaction sites in the tertiary structure of the studied proteins. We posit that the network of various contacts established and/or eliminated during the evolution of alternate conformational rearrangements in the family of allosteric proteins forms an essential part of the eventual development of diversity in individual protein function. Accordingly, multiple sequence alignment (MSA) (Hogeweg and Hesper 1984) has also become a powerful computational tool for uncovering the long-term evolutionary record of a protein family. Therefore, the interdependence of evolutionary history or coevolution, which can be obtained from MSAs, is also used in this work as a means to predict intermolecular communication between residue pairs and hence, allosteric coupling that is related to protein functionality.

Using this multimethod approach we contrast three structurally similar proteins in the c-type lysozyme family (equine lysozyme [EL], α-lactalbumin [LALBA], and conventional lysozyme) and demonstrate that sequence differences that alter the equilibrium of conformational ensembles are also the basis for the acquisition of distinctive function in the representative structures. Specifically, comparison of the intrinsic dynamics of the native state of the two calcium-binding proteins LALBA and EL indicates that the observed differences in collective fluctuations also form the foundation for functional divergence. Furthermore, when considering the antimicrobial properties of protein–oleic acid (OA) (EL–OA and HAMLET [Human α-lactalbumin Made LEthal to Tumor cells]) and protein–oligosaccharide (lysozyme–(NAG)_3_ [tri-*N*-acetyl-d-glucosamine]) complexes of individual proteins in the family we find that ligand sensitive equilibria adjust allosteric interactions that appear to have a central role in target specificity. Altogether, these findings suggest that conformational dynamics may play an important role in the mechanism in which protein function evolves through novel molecular interactions. (table 1).

**Table 1.** Frequency and Description of the Motions of the Labeled Peaks in the Experimental THz Spectrum in Figure 7.

Wavenumber (cm^−1^)	Description
115	Torsional motion of A/B loop
130	Protein mainchain–solvent H-bonding torsion
135	Side-chain torsion from protein–protein interactions in hydrophobic core
146	Protein backbone–solvent-coupled mode(structured water)
153	Protein backbone–solvent-coupled mode(unstructured water)

## Results and Discussion

### The Evolution of Conformational Ensembles and the Divergence in Protein Dynamics: EL and LALBA

#### Experimental Detection of Protein Intrinsic Fluctuations in Response to Ligand-Binding

**Fig. 1.**
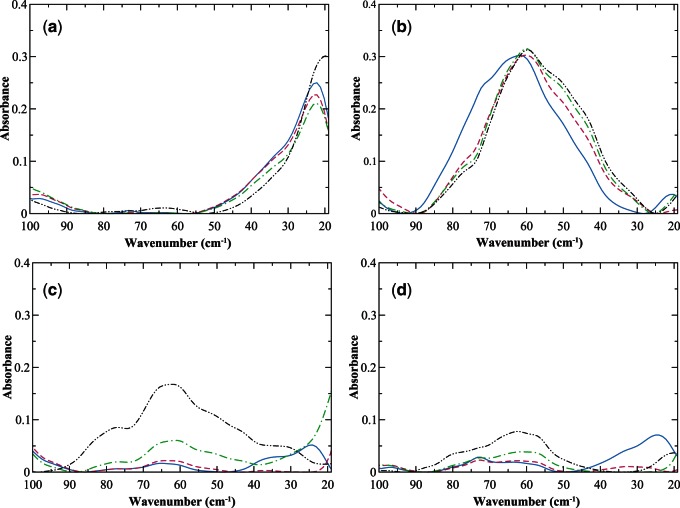
Experimental THz spectrum of (*a*) EL-Ca^2+^, (*b*) MG-LALBA, (*c*) LALBA-Ca^2+^, and (*d*) HAMLET in the 20–100 cm^−1^ spectral region at 93 K (solid, blue line), 150 K (magenta, dotted line), 200 K (green, dotted-dashed line), and 300 K (black, dotted-dotted-dashed line).

When contrasting the experimentally detected intrinsic dynamics of EL and LALBA when bound with calcium as shown in figure 1*a* and *c*, it becomes apparent that there are noticeable differences in the detectable low-frequency fluctuations of the two proteins. In figure 1*a*, the experimental THz spectrum of EL has a single, prominent peak close to 20 cm^−^^1^. Based on both the frequency and the temperature dependency of this prominent mode, we deduce that it arises from torsional motion of protein side-chain fluctuations (Woods 2010). It is interesting to point out that the experimental low-frequency map of global fluctuations in EL is comparable to that of calmodulin, another calcium-modulated protein (supplementary material S2, fig. S2, Supplementary Material online). In fact, previous computational studies on calmodulin have indicated that torsional motions rather than large-scale backbone motion are the primary source for propagating signals over long-length scales in the protein (DuBay et al. 2011). So based on the experimentally detected similarity of global motion fluctuations in the two proteins (calmodulin and EL), it is probable that torsional oscillations also play a prominent role in the signal propagation process in EL.

Comparing the experimentally observed molten globule (MG) state of EL with that of the native state reveals very few distinctions (not shown). In stark contrast, the experimentally observed MG state of LALBA in figure 1*b* differs remarkably from its native state in figure 1*c*. In figure 1*b*, we notice that the experimentally detected global dynamics of the MG state of LALBA is dominated by a composite of thermally induced side-chain fluctuations that are centered at 60 cm^−^^1^ and a small peak at approximately 20 cm^−^^1^, none of which change appreciably as a function of temperature. While on the other hand, the native state of LALBA in figure 1*c* possesses a combination of backbone and side-chain fluctuations that are most apparent above the protein transition temperature. At the lowest temperature investigated, there are visible peaks at approximately 63, 37, and 23 cm^−^^1^ in the calcium-bound LALBA sample (fig. 1*c*) and a smaller shoulder located at approximately 80 cm^−^^1^. The approximately 80 and 63 cm^−^^1^ bands are attributed to backbone deformations and intraprotein-induced fluctuations within the protein core whereas the 37 and 23 cm^−^^1^ peaks are associated with solvent-exposed and nonpolar side-chain fluctuations, respectively. As the protein progresses through the transition temperature, both the 80 and 63 cm^−^^1^ bands continue to grow in amplitude whereas the side-chain fluctuations have a more complex pattern that require some consideration to discern. The 37 cm^−^^1^ mode decreases in intensity as the temperature proceeds through the transition temperature and the 23 cm^−^^1^ mode shifts to a lower frequency that is outside of the experimental window. Above the transition temperature, a new mode appears at approximately 30 cm^−^^1^ and this is attributed to delocalized (surface) side-chain fluctuations that permeate throughout the entire protein structure (Diehl et al. 1997; Paciaroni et al. 1999; Joti et al. 2008; Woods 2014a).

Experimentally, the less than 100 cm^−^^1^ region of the infrared spectrum is sensitive to the global, internal fluctuations that define the intrinsic dynamics (Tobi and Bahar 2005; Bahar et al. 2007; Meireles et al. 2011) of proteins. These globally, correlated fluctuations allow the protein to sample the ensemble of conformations that govern the free energy landscape of all possible protein conformations (Xu et al. 2006; Heyden et al. 2010; Ding et al. 2012). It has been suggested that changes in protein global motions in response to extrinisic perturbation, such as ligand-binding, provide a means of determining how sampling of the available protein conformational substates shifts the distribution of propagation pathways in the protein structure (Frauenfelder et al. 1991; Meinhold et al. 2007; Ishikawa et al. 2008; Okazaki and Takada 2008; Nussinov and Tsai 2013). These external disturbances are transmitted as modifications in the population of available protein conformations that are sampled and as a result can dramatically influence overall protein function. Hence, allostery is a direct result of the redistribution of protein conformational ensembles (Fenwick et al. 2011). Despite the surge in interest in mapping conformational ensembles to protein function, structural characterization of the populated states in the ensembles has been historically difficult to detect with experimental measurements (Fraser et al. 2011; Motlagh et al. 2014). THz spectroscopy provides unique, direct information about the collective, global fluctuations (Zhang et al. 2004; Xu et al. 2006; Ding et al. 2011; He et al. 2011; Zakaria et al. 2011; Woods 2014a) in proteins that is not accessible by many, traditional spectroscopy methods. Therefore, it offers a novel tool for probing the intrinsic fluctuations in proteins and at the same time has the potential to provide innovative insight into the formation of signaling pathways in allosteric proteins and enzymes.

For instance, both LALBA and EL are examples of mammalian milk proteins in the c-type lysozyme subfamily that bind calcium (Calcium-binding is known to induce conformational changes in other calcium-binding proteins; which in turn, are connected with enhancing interactions with other biological macromolecules.) in their native, functional state. Although sharing strong structural similarity and moderate (35–40%) sequence identity (Iyer and Qasba 1999), LALBA and EL have entirely different functions. Calcium-binding in LALBA has a structural role. When not bound by Ca^2+^, LALBA adopts a partially folded stable structural intermediate known as the molten globule or MG state (Iyer and Qasba 1999; Chrysina et al. 2000). The MG state has native-like secondary structure but lacks the packing interactions necessary for stabilizing the tertiary structure of the protein. Binding calcium stabilizes LALBA into one of its possible conformations and through this functional conformation subsequently initiates binding to galactosyl transferase (GT) to eventually produce lactose. EL also assumes an MG state when not bound with calcium, but in this case the folding intermediate is far more stable when compared with calcium-free LALBA (Morozova-Roche et al. 1997). Where the functional conformation of EL is assumed to be associated with hydrolyzing peptidoglycan components in bacterial cell walls.

It has been suggested that there is a relationship between the lowest energy global modes and the routes through which protein structures diverge through mutation (Echave and Fernández 2010). Particularly in this context it is very probable that LALBA and EL represent examples of proteins that share a common ancestor (Prager and Wilson 1988; Irwin et al. 2011) yet are functionally divergent due to mutations that have shifted the equilibrium of ensembles away from an original, ancient set of ordered structures. In this framework, a mapping of variations within the intrinsic (global) motions of the individual proteins in a family may be useful in both tracing the origin of and predicting functional divergence.

#### Correlation Analysis to Determine the Intrinsically Favored Conformational States of Calcium-Bound EL and LALBA from MD Simulation

**Fig. 2.**
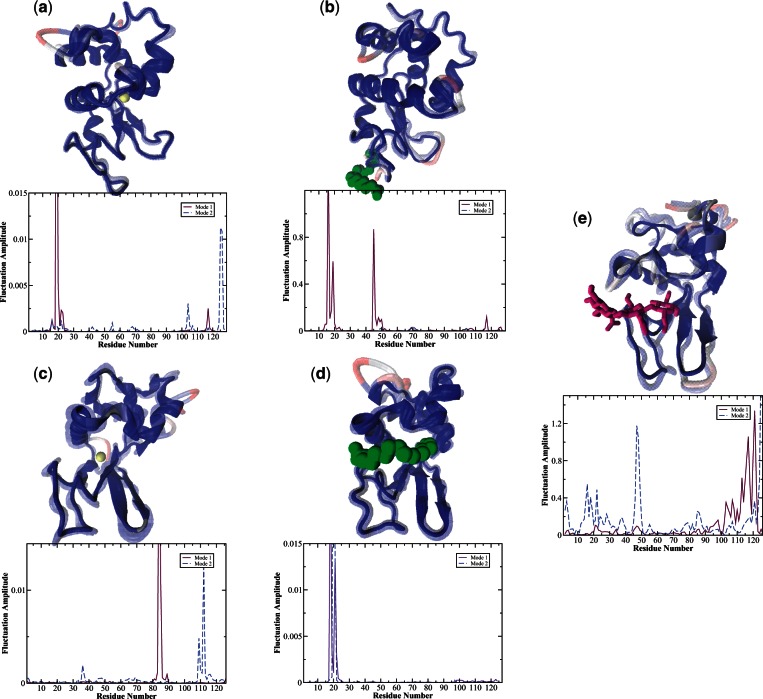
Projection of the two dominant modes from the correlation analysis is mapped onto a cartoon representation of the protein structure of the protein–ligand system. In the representation, the ligand is drawn as either van der Waal radii or depicted in a licorice representation in (*a*) El-Ca^2+^, (*b*) EL-OA, (*c*) LALBA-Ca^2+^, (*d*) HAMLET, and (*e*) LYZ-(NAG)_3_. In the cartoon representation of the protein, red areas of motion reflect regions of higher mobility, whereas areas in blue reflect lower mobility. In the graph below the 3D representation of the protein–ligand system, the weighted square displacements (using LYZ numbering) of the C-α residues in the protein along the most anharmonic mode (solid purple line) and second most anharmonic mode (blue dashed line) are shown.

**Fig. 3.**
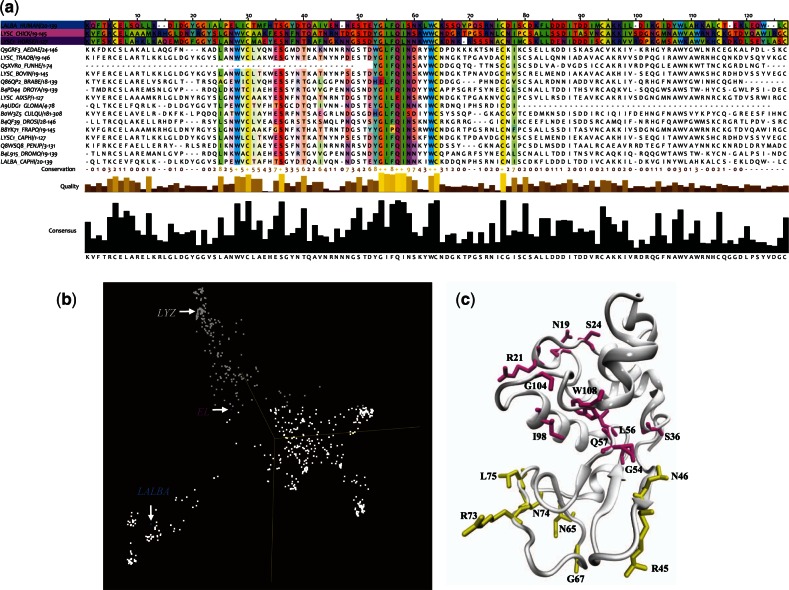
(*a*) Snapshot of the MSA visualized with jalview (http://www.jalview.org/) showing a small number of the aligned sequences. Representative sequences described in this article are highlighted in blue, gray, and purple. The highlighted MSA sequence residues are colored with the Taylor coloring scheme. The other sequences shown but not highlighted also feature the Taylor coloring method but only of conserved residues. The middle and bottom panels of the MSA visualize the conservation of the aligned sequences as histograms and a score for each column. A score of “*” indicates that the amino acid is maximally conserved and a score of “0” indicates that the amino acid is minimally conserved. (*b*) PCA of the MSA calculated using the BLOSUM26 score matrix depicting a spatial representation of the similarities of all of the sequences in the alignment. The set of sequences is displayed such that similar sequences lie near to each other in 3D space. The sequences discussed in the text of the manuscript are highlighted in blue, gray, and purple in the spatial representation. (*c*) Cartoon representation of the reference structure from the MSA (1LYZ.pdb) and sequence evolution analysis. Residues with the highest MI values in the α-domain are drawn with a licorice representation and colored magenta whereas those in the β-domain are drawn with a licorice representation and colored yellow.

In addition to experimental measurements, we have also used correlation analysis from MD simulation to quantify concerted fluctuations taking place in the c-type lysozyme sequences investigated in this study. From this type of analysis, it is possible to refine the overall description of how changes in the intraprotein interactions influence the collective fluctuations taking place within the protein that are not readily available from experiment. For instance, a map of the residue correlations of EL bound with calcium in figure 2*a* from correlation analysis reveals regions in the protein secondary structure that support concerted fluctuations. The projection of the correlated motions onto the protein structure (fig. 2*a*) clearly demonstrates the effect of the fluctuations on the dynamics of the entire protein. Particularly, when the calcium ligand is bound, the greatest motion is remote from the actual ligand-binding site. Instead, the response from ligand-binding is concentrated almost exclusively in localized regions in the α-domain with only a limited contribution from residues in the β-domain. Binding causes a hinge-like torsion in the vicinity of the calcium-ion binding spot. Particularly, the perturbation triggered by ligand-binding is alleviated by localized torsional motion mainly involving Leu 83, which is directly adjacent to the ligand-binding site. The external disturbance caused by ligand-binding is further propagated through the interior of the molecule by small, rotational fluctuations involving core residues, principally Ser 36, Gly 54, Leu 56, Gln 57, Gly 104, and Trp 108 (fig. 2*a* and supplementary material S7, fig. S7*a*, Supplementary Material online). These core residue fluctuations are transmitted through the protein interior and disseminated outward toward exposed loop regions and ultimately to surface residues that reside opposite the ligand-binding site. These small-amplitude variances in the protein core are coupled with large-amplitude fluctuations taking place on the protein surface. The core residues that are activated in response to ligand-binding are for the most part conserved (fig. 3*a*) and represent an optimized network of intraprotein interactions that undergo small-amplitude rotational oscillations that mandate stability within the protein interior. Meanwhile the amino acid groups that are ultimately excited on the protein surface, chiefly Asn 19, Tyr 20, and Lys 116, are comprised residues that are not conserved but encompass parts of the A/B loop as well as portions of the 3_10_-helix in the α-domain.

In contrast to EL, calcium-induced correlated fluctuations in LALBA lead to a substantial increase in interdomain coupling. It is known that calcium-binding in LALBA affects packing at the subdomain interface, which in turn leads to structural changes that are most prominent on the opposite side of the calcium-binding ligand site (Iyer and Qasba 1999; Vogel 2002). This mechanism involves a shift in interresidue dynamics that subsequently channels fluctuations away from the ligand-binding site. Consequently, this transference is directly intertwined with overall protein function (Anderson et al. 1997; Kuhlman et al. 1997; Chrysina et al. 2000; Svensson et al. 2000). From the MD simulations carried out in this study, we observe that the cluster of (calcium) binding-site residues in LALBA has much greater translational mobility when compared with that of EL-Ca^2+^ (fig. 2*c*). In particular, the mobility of Leu 84 has a central role in directing excess binding energy away from the ligand-binding site and funneling it to distant regions in the protein 3D structure (supplementary material S7, fig. S7*c*, Supplementary Material online). Analogous to EL-Ca^2+^, fluctuations of binding-site residues influence the dynamics of the A/B loop of the protein but in this case the effect is greatly diminished in the localized A/B loop region. Instead, the dynamical motion of the ligand-binding residues has greater impact on the overall protein structure. For instance, the large-amplitude oscillations of Leu 84 direct the ligand-binding loop inward toward the anti-parallel β-sheet, thereby also activating hydrophobic contacts in flexible, exposed regions of the protein.

Also significant is that the pathway to surface residue excitation in LALBA- Ca^2+^ appears to differ significantly from EL-Ca^2+^. The most distinguishable difference is that the strain induced from ligand-binding in LALBA is transmitted through the surface of the protein rather than through the protein hydrophobic core. This modification in the energy transfer mechanism is attributed to sequence mutations adjacent to the active-site cleft and modified hydrogen-bonding interactions (Anderson et al. 1997; Iyer and Qasba 1999; Chrysina et al. 2000) at the intersection of the α− and β−domain that are activated with ligand-binding (supplementary fig. S7*c*, Supplementary Material online). As a result, the hydrophobic core is rather rigid and interdomain coupling is maximized in calcium-bound LALBA. The interdomain coupling that accompanies calcium-binding is also the foundation for the formation of large-amplitude, localized fluctuations on the protein surface. Namely, collective bending torsions at the intersection of the α-/β-lobe (that develop as a result of calcium-binding) overlap with the dynamics of flexible long-loop residues (residues 60–80) in the β-domain that are jointly coupled with the dynamics of a cluster of residues in the α-domain. The residues excited in the α-domain include amino acids in Helix D, the 3_10_-helix, and residues forming the aromatic cluster I (Grobler, Wang, et al. 1994) on the protein surface. Together, the collective dynamics that are activated at the intersection of the domains culminate as an accordion-like fluctuation centered in a localized region on the protein surface (fig. 2*c*). It is interesting to point out that the excitation of residues in both Helix D and the 3_10_-helix that we have uncovered from our computational investigation on the collective dynamics of LALBA-Ca^2+^ has also been detected in previous experimental measurements (Grobler, Wang, et al. 1994) on the calcium-bound protein. In these earlier measurements, the detected surface fluctuations were associated with establishing the contact region for GT binding during lactose synthase activity.

#### Summary: Intrinsic Dynamics and Evolution of Protein Function in Calcium-Binding Lysozyme and Mammalian LALBA

As mentioned previously, allosteric mechanisms are interrelated with the relative stabilities of conformational substates. Consequently, allostery is a dynamic process that is described by an equilibrium of ensembles (Volkman et al. 2001; Kern and Zuiderweg 2003). And in proteins, shifts in these ensembles have evolved in order to acquire specific, functional roles. Furthermore, the results of the experimental measurements carried out in this investigation suggest that the binding-induced conformational changes in the two different milk proteins (LALBA and EL) may illustrate the effect of mutation on the evolutionarily conserved networks of the protein family and accordingly, the direct influence of the evolution of intrinsic modes on the properties of the individual proteins that comprise the family. In this interpretation, modifications within the available conformations are expected to lead to variations in the globally encoded intrinsic fluctuations that ultimately map out protein function. Therefore, it is likely that the variances in the network of concerted fluctuations that we have observed both experimentally and computationally in LALBA and EL (figs. 1*a*–*c*, 2*a*, and 2*c*) are directly intertwined with deviations in the conserved interactions that direct the sampling of the functional conformations within the ensembles of the two structurally similar proteins and at the same time may define their distinct functions.

### MD Simulation, MSA, and Coevolved Networks in the c-Type Lysozyme Family

#### MSA in the Mammalian c-Type Lysozyme Family

If MSA of a protein family is large and diverse enough, it describes the evolutionary characteristics of that family (Liu and Bahar 2012). To understand the relationship between conserved and adaptable characteristics in the c-type lysozyme family, we have utilized network analysis to assess trends in sequence evolution. Native ensembles of proteins within the same family provide information about the conserved, instrinsic dynamics within the set of conformations. Therefore, a hierarchical clustering of top ranking modes conveys information about the most accessible conformations within the ensemble of functional structures (Nussinov and Tsai 2013).

MSAs of structurally similar proteins carry two distinct forms of information. The first type, which has already been discussed, is the conservation of amino acids at certain positions in the protein family. The other is the interrelationship between two or more positions in the evolutionary network. Mutual information (MI) from information theory can be used to infer the extent of the coevolutionary relationship between two positions in a protein family (Simonetti et al. 2013). For this reason, MI is often applied to predict positional correlations in an MSA with the intention of guiding the identification of structurally or functionally important positions in a given protein family. Highly conserved positions identify residues whose mutations are likely to disrupt overall protein function (del Sol et al. 2006), whereas compensatory mutations often occur and are essential for the adaptation of new functional characteristics (Morcos et al. 2014). Proteins that evolve from a common ancestor can change functionality over time. Therefore, protein functional dynamics can be considered as an evolving, landscape created by the interplay between the global, collective fluctuations encoded in the protein structure and the localized protein interactions that define specificity (Romero and Arnold 2009). Therefore, long-range communication is embedded in the evolutionary record of a protein family. And trends in correlated mutations in aligned sequences have the potential to reveal conserved allosteric signal pathways.

**Fig. 4.**
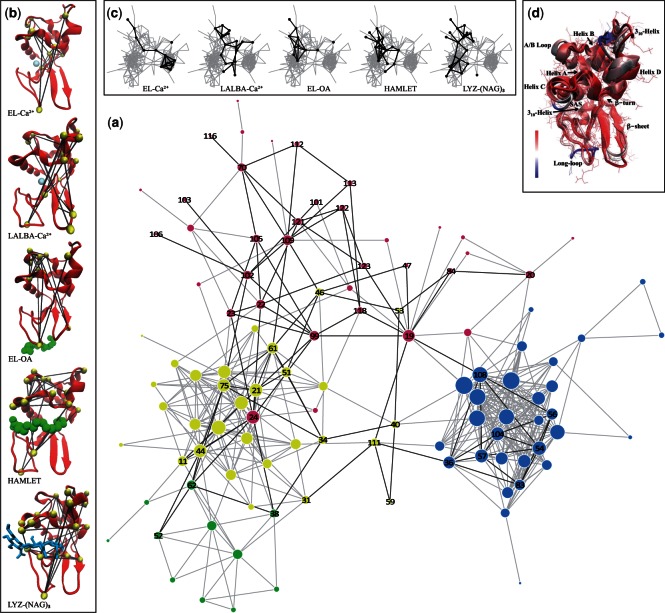
(*a*) Network representation of the MSA where the nodes represent amino acids with numbering from the reference structure (1LYZ.pdb), the size of the nodes represent the score of the MI values from the sequence evolution analysis of the MSA, and the color represents their respective network communities. The positions of the nodes result from a two-step process of classical scaling and stress minimization (Brandes and Pich 2009). In the network representation, only the most important nodes (as determined from the correlated fluctuations uncovered from the correlation analyses) from the protein–ligand systems studied are labeled and the edges between the important nodes are drawn with dark gray lines. (*b*) The images depict a network representation of the most important nodes of the selected protein–ligand system mapped onto a cartoon representation of the protein with the ligand depicted with either van der Waal radii or a licorice representation. (*c*) Overview picture of the coevolutionary network from the MSA highlighting important residues and their connections in the protein–ligand systems. The images emphasize the regions of the network that are utilized by each individual protein–ligand system. (*d*) Overlay of the three protein sequences studied in this work with the secondary structural regions labeled. The molecules are colored by their structural identity. The red areas indicate that the molecules are structurally conserved at those regions, whereas the blue indicates more structural difference in the corresponding regions.

In this investigation, an ordered clustering of residues with significant MI values from the MSA has revealed that many of the residues with the greatest values lie within the protein hydrophobic core (figs. 3*a*–*c* and 4). Likewise, many of these high MI value residues are both moderately conserved in the alignment and also have significant dynamical correlation between them. For instance, the strongly coupled residues (Note that the numbering of residues in all of the proteins discussed in this manuscript is based on the numbering of the hen egg white lysozyme [HEWL] reference structure 1LYZ.pdb.) Gln 57, Gly 104, and Trp 108 are all close in proximity and their correlated dynamics play an important role in maintaining stability (Greene et al. 1999; Ye et al. 2007) in the structurally similar proteins included in the alignment. A computational study (Wohlkönig et al. 2010) on structural relationships in the greater lysozyme superfamily has revealed a family-specific sequence motif containing a common beta-hairpin structure. Within the conserved structure there is a high degree of conservation for particular residues at specific positions in the hairpin, mainly Gly 54, Leu 55, Gln 57, and Ile 58. The conservation of these residues in the hairpin structure indicates a requirement for a specific conformational or functional role in the enzymatic function in the general lysozyme family. It has been suggested that Leu 55, Gln 57, and Ile 58 are involved in protein stability, whereas the flexibility of Gly 54 is believed to play a crucial role in the shared catalytic mechanism utilized by enzymes in the superfamily (Wohlkönig et al. 2010).

#### Mapping of the MSA onto EL Correlated Dynamics from MD Simulation

We integrate coevolutionary trends from the phylogenetic analysis with structural variations in dynamics (as deduced from MD simulation) to develop a deeper understanding about the relationship between evolution and conformational selection in the proteins investigated. From the correlation analysis outlined in the previous section, we have determined that ligand-binding disrupts the stability of the hydrophobic core in EL and this subsequently leads to localized mobility on the protein surface. Mapping the collective dynamics from the MD simulation onto the MSA network (fig. 4*b*) makes it evident that the residues establishing the most efficient pathway in EL, in response to ligand-binding, are comprised a combination of conserved residues joined with residues with intermediate MI values (fig. 3). Specifically, rearrangement of aromatic side-chains of residues Val 99, Met 105, and Pro 70, which form part of the exposed loop region, induces slight structural variations within the protein interior. The perturbation from ligand-binding is further propagated through the interior of the molecule by small, rotational fluctuations comprising conserved core residues Gly 54, Leu 56, Gln 57, Gly 104, and Trp 108. Together the coupled set of residues functions to form an effective pathway through the protein hydrophobic interior.

It is also worth pointing out that in addition to the sequence-dependent optimal pathway detected in EL, there are many other preexisting pathways of communication that are resolved from the MI analysis that may also have a functional role in the c-type lysozyme family. Community analysis (Girvan and Newman 2002; Newman 2004) reveals four distinct sequence-dependent pathways for communication in the alignment of the sequences (fig. 4*a*). In communities, nodes within the same community are highly connected and communicate with one another very efficiently but often in a localized manner, whereas nodes in different communities have fewer connections and require intermodular edges (or links) for creating long-distance or intercommunity communication (Newman 2006). Residues that form these links between communities are capable of altering a pathway from a suboptimal to an optimal path by establishing channels of communication within the protein network (del Sol et al. 2006). Furthermore, alterations to established networks are a necessary precursor for the adoption of new functional properties in a protein family (del Sol et al. 2006). In this respect, residues that connect distant regions in the protein are composed of two distinct types of amino acids: Highly conserved residues that are centrally located in the protein core that preserve the structure of the shortest, most effective pathways and residues with high coevolution propensity that mediate enhanced signaling in the network. It is through these “linker” residues that a pathway is modified when a perturbation is introduced and their role in mediating specificity in molecular recognition has also been demonstrated (Giliani et al. 2002; Hill et al. 2002; Binkowski et al. 2003; Liu and Bahar 2012).

For instance, in EL-Ca^2+^ the dynamic interaction between Trp 108 and Asn 19 forms links between two distinct communities (fig. 4*a*) that appear to facilitate long-distance protein intermolecular communication. Although in calcium-bound EL, long-range communication in the protein seems to be rather limited. Tightly packed regions in the protein interior undergo localized fluctuations when bound by calcium, producing coherent fluctuations on the protein surface. And the residues most affected on the surface are high MI residues making up the flexible regions of the A/B loop (fig. 4*b* and *c*). The formation of the confined surface fluctuations is also the principal source of collective dynamics in the ligand-bound protein. But even in this case only a small number of residues have a prominent role in the development of these restricted, collective surface fluctuations. The formation of the collective oscillations in EL-Ca^2+^ is linked with the overlap of dynamics among residues in the protein hydrophobic core and residues distantly located on the protein exterior. And both Trp 108 and Asn 19 have a prominent role in the development of the ligand-induced coherent fluctuations. Incidentally, our computational analyses of EL-Ca^2+^ are in line with our experimental observations of the global modes of the ligand-bound protein in figure 1*a*. Where experimentally, we also observe that EL has very limited collectivity associated with the detected global modes.

#### Mapping of the MSA onto LALBA Correlated Dynamics from MD Simulation

Experimental measurements (fig. 1*c*) and computational modeling (fig. 2*c*) of LALBA global motions indicate that the protein acquires a substantial increase in interdomain coupling when bound with calcium. As a result, there is an increase in the extent of collective fluctuations that develop in the protein 3D structure and also an altered mechanism of signal propagation due to the perturbation of ligand-binding. Both features of LALBA-Ca^2+^ differ significantly from EL-Ca^2+^. We attribute the variations in domain coupling and signal propagation to alterations in protein sequence and a subsequent shift in the intraprotein interactions at the subdomain interface. Mapping of the MSA onto the correlated dynamics of calcium-bound LALBA provides greater insight into the activated mechanics of the interdomain dynamics as well as the underlying source of the ligand-induced signal propagation mechanism. For instance, in LALBA-Ca^2+^ the dynamic link between calcium-binding residue Leu 84 and β-sheet residue Tyr 53 is instrumental in the mechanism associated with interdomain coupling (fig. 4*a* and supplementary material S7, fig. S7*c*, Supplementary Material online). Both residues belong to distinct communities and their association creates a connection between stable, structure-forming regions within the protein core and a high coevolution propensity region (Leu 84) that facilitates enhanced long-range signaling in the network through large-amplitude translational motion (fig. 2*c*). Similarly, the dynamic correlation between residues Asn 46 and Val 109 is associated with collective fluctuations that assist in the mechanism of intraprotein signal transduction. Asn 46 is also a β-sheet residue, but unlike Tyr 53 it has high sequence variability in the structural alignment and possesses moderate conformational mobility in the global modes (figs. 2*c* and 3*a*–*c*). The correlated fluctuations formed by the intersection of the two network communities (containing Asn 46 and Val 109) ultimately create a delineated network of intermolecular associations on the protein surface (fig. 2*c*). The extended surface excitations are concentrated primarily in the α-domain and include high mobility residues comprising regions in the 3_10_-helix along with aromatic residues adjacent to the active-site cleft (aromatic cluster I) (Grobler, Wang, et al. 1994).

#### Summary: Sequence Evolution and Protein Dynamics in EL and LALBA

This approach of highlighting the connection between the concerted fluctuations taking place in calcium-bound EL and the sequence patterns from the MSA clearly demonstrates that ligand-binding disrupts the stability of a set of residues that are conserved in the family, which subsequently leads to localized mobility on the protein surface. Particularly, coevolved residue pairs within the A/B loop make the greatest contribution to the surface-induced fluctuations. It is possible that this localized flexibility in the protein interior, coupled with the prominent motion of the A/B loop, has some role in orientating the substrate into the binding cleft or perhaps assisting in substrate recognition. The residues excited on the surface of EL in response to ligand-binding are documented antigenic sites (Atassi et al. 1976; Padlan et al. 1989) in conventional lysozyme. So it is plausible that the confined mobility that develops from calcium-binding would enhance complementarity between that antigen and receptor at points along the antigenic surface.

In LALBA, it has previously been established that only in the native state (bound with Ca^2+^) is the protein able to associate with GT (Grobler, Wang, et al. 1994; Kuwajima 1996; Anderson et al. 1997). The activation of the protein in the presence of its native ligand is useful for understanding the functional evolution within the c-type lysozyme family in addition to the evolution of protein interactions within the family. The structural pathways formed by collective fluctuations that originate at the binding site are drastically different in EL-Ca^2+^ when contrasted with LALBA-Ca^2+^. And perhaps most unexpected is that EL and LALBA use the hydrophobic core in entirely different manners. In EL, only a small subset of residues are involved in the ligand-induced signal transmission process. Particularly, in calcium-bound EL a few high-mobility residues are linked with signal transmission whereas LALBA utilizes high stability regions and domain coupling to promote long-range protein communication that supports an extended network of collective fluctuations. Moreover, perturbation of the evolutionarily conserved network of amino acid interactions in the vicinity of the domain interface appears to underlie the shift in interresidue interactions and hence, the appearance of new functional properties in LALBA. The adaptations in sequence in the interdomain region excite an extensive array of surface fluctuations in LALBA that have previously been identified as a functional region associated with substrate recognition and stabilization (Grobler, Wang, et al. 1994; Greene et al. 1999; Chrysina et al. 2000; Jøhnke and Petersen 2012).

### Connection between the Evolution of Protein Interaction Networks and the Progression of Antimicrobial Properties in c-Type Lysozymes

#### The Relationship between the Immune Response to Infection and the Origin of Lactation in Mammals

The mammary gland is unique to the class Mammalia and its role is to provide newborns with milk. Breast milk has both nutritional and immunological properties. It has been associated with regulating the early phase of gut colonization by microorganisms as defense in infant immunity (Oddy 2002; Hanson et al. 2003). For instance, breastfed babies have a lower incidence of gastrointestinal infection compared with their formula-fed counterparts. It has been hypothesized that lactation evolved as an inflammatory response to tissue damage and infection as part as the evolution of the innate immune system (Vorbach et al. 2006). In this view, the ancient mammary gland initially developed as organs that produced large amounts of antimicrobial factors to protect evolving mammalian skin. And during this evolution process, lysozyme evolved a dual role in the lactating mammary gland: 1) An original antimicrobial role and 2) later, after gene duplication, a reproductive role that eventually leads to the production of LALBA and the regulatory effect of LALBA in the formation of lactose synthesis. In summary, the nutritional role of milk evolved subsequent to its protective function. And in this interpretation, lactation evolved incrementally in steps. So one of the subject matters that we attempt to address is whether lactation and the inflammatory response of the structurally similar proteins share a common mechanism that can be traced back to the evolution of their inherited protein evolutionary networks. We begin with the premise that the genes involved with antimicrobial properties in the mucous membrane went through gene duplication and evolved additional roles in the mammary epithelium. Hence, the molecules controlling inflammatory responses were key regulators and necessary triggers for the evolution of modern lactation. One of the genes involved in evolving mammalian skin is the current *LYSC1* gene of which modern day EL is a member. The *LYSC1* gene has been found to be secreted as an inflammatory response in tissue as a reaction to a wide range of infections (Waterhouse et al. 2007; Kajla et al. 2011). Therefore, it is conceivable that understanding the intermolecular interactions that are excited in EL in the connection with its antimicrobial immune activities may also provide insight into the evolvement of analogous but distinct associations in the antibacterial defense mechanisms of structurally similar proteins in the same family.

#### Evolutionary Dynamics of Coevolutionary Links and the Antimicrobial Properties of c-Type Lysozymes Explored with MD Simulation and Sequence Evolution Analysis

##### Computational Analysis of Correlated Fluctuations and Dynamics of Coevolutionary Links in EL Complexed with Oleic Acid.

There have been a number of investigations on the cytotoxic activity of proteins in the lysozyme family when complexed with lipid molecules found naturally in milk. In fact, several recent experimental investigations on EL (Morozova-Roche 2007; Wilhelm et al. 2009; Clementi et al. 2013) have uncovered that the protein bound with OA (EL-OA [EL complexed with OA]) possesses bactericidal activity that shares remarkable similarities with the (more familiar) human lysozyme counterpart complexed with OA, HAMLET (Marks et al. 2012). Although, HAMLET is better known for its targeted cytotoxic activity toward mammalian cancer cells (Svanborg et al. 2003; Kamijima et al. 2008; Pettersson-Kastberg et al. 2009; Mercer et al. 2011; Ho CS et al. 2012; Wen et al. 2013) than its interaction with bacteria.

From this investigation, we find that MD simulation of EL-OA complexes indicates that EL dynamics is not dramatically altered when bound to OA (fig. 2*b*). OA preferably binds near Pro 70 in the long-loop region of the β−domain and this slightly modifies the dynamic coupling between the two, separate domains of the protein. Specifically, binding of OA moderately increases the amplitude of motion of residues within the β-sheet while at the same time promoting modest rigidity within the hydrophobic core. A look at the graphical representation of the most prominent modes from the MD simulation of EL-OA in figure 2*b* reveals that certain regions in the protein structure are visibly enhanced with OA ligand-binding and differ somewhat from that of the protein in its native state (fig. 2*a*).

Combining the evolutionary relationships extracted from the MSA with the global, correlated protein dynamics determined from MD simulation, we observe the prompting of principal (coevolutionary) links between two high MI residues that foster enhanced dynamics associated with substrate binding and interdomain coupling (figs. 2*a* and 4). In particular, Ser 24 in Helix B of the α-domain has correlated dynamics with Arg 45 in the β-domain antiparallel β-sheet. The reorientation dynamics resulting from the correlated motion from the two distinct regions (communities) leads to increased localized fluctuations on the surface of the protein that are mainly clustered about the A/B loop, Helix B, and a limited region of the 3_10_-helix in the α-domain.

It is interesting to mention that more recent investigations on EL-OA have indicated that when the protein-OA complex is bound to bacterial membranes, specific association with the surface of the extraneous cells promotes membrane defects and eventual cell lysis (Clementi et al. 2013). In other words, the cell membrane is the primary target for EL-OA toxic activity. From our computational investigation on EL-OA, we find that the regions of the protein that are excited with OA binding are strongly reminiscent of the antibody–antigen structure of native (conventional) lysozyme (Padlan et al. 1989). As part of the adaptive immune system, antibodies that bind on cell surfaces mediate cell–cell recognition and/or interact with other molecules involved in intracellular signaling. Many of the known lysozyme antibody-antigenic association subsites closely correspond with the residues in EL that are activated as a result of binding to OA in this study.

Previous experimental studies (Wilhelm et al. 2009; Clementi et al. 2013) have indicated that EL-OA is highly effective in terms of its targeted, bactericidal activity at low concentrations but has been found to be indiscriminately cytotoxic to mammalian cancerous cells as well as healthy, differentiated mammalian cells at an equivalently low concentration. Therefore, it is plausible that the nature of the conformational changes occurring in EL after complex formation with OA underlies the targeting mechanism associated with both recognition and subsequent cytotoxic membrane properties. From the MD simulations carried out in this investigation, we do observe a slight modification in the surface properties of EL when bound to OA that differ from the protein in its native state. It is possible that these modifications relate to its effectiveness as an antimicrobial agent.

##### Computational Analysis of Correlated Fluctuations and Dynamics of Coevolutionary Links in HAMLET.

Exploring HAMLET dynamics with MD simulation reveals that binding of OA in the protein–ligand complex produces side-chain rearrangements of residues in the active-site region that have distant effects on surface residues in the α-domain. Specifically, binding of the ligand produces anharmonic fluctuations of the active-site residue Met 105 and this causes slight structural changes and packing interaction modifications within the protein core (fig. 2*d*). Met 105 is a pivotal residue that divides the tightly packed, rigid core of the protein from surface flexible loop regions in the α-domain (fig. 4). Accordingly, hydrophobic contacts between the ligand and Met 105 play a prominent role in determining the conformation of the ligand in the enzyme complex. Analogous to what we have observed in native EL (El-Ca^2+^), the rearrangement of amino acid side-chain fluctuations in the vicinity of Met 105 is communicated through mechanical force as excitations that affect residues on the surface of the protein (supplementary material S3, figs. S4 and S7*d*, Supplementary Material online). And ultimately the disruption of interactions in the protein core results in enhanced, localized, rotational mobility that is most prominent in the region corresponding to the A/B loop but in this case also includes residues making up the solvent-exposed loop (residues 102–106) as well as residues associated with the aromatic cluster I in native LALBA, to a lesser extent (fig. 2*d*).

It is interesting to mention that in our simulations we find that OA (in HAMLET) preferentially binds in the protein’s catalytic binding cleft. In this orientation, Arg 61, which is located at the edge of the active-site cleft, couples with the dynamics of OA by means of hydrophobic interaction (fig. 2*d* and supplementary fig. S3, Supplementary Material online). The coupled ligand–protein interaction has an effect on both the local and the global dynamics of the protein. The induced dynamics from the coupling propagates outward from the binding site toward surface residues that adjust both the stability and positioning of flexible loops on the protein exterior (supplementary material S7, Supplementary Material online). Namely, the collective, torsional fluctuation activated by the protein–ligand interaction extends to residues Val 109 and Ala 110 on Helix D and Phe 34 on Helix B. Overall the presence of OA in the LALBA active-site culminates as a restricted hinge torsion that is localized mainly in the α-domain of the HAMLET complex, with the largest motion concentrated about the A/B loop (figs. 2*d* and 4*b*).

The larger surface area excited in HAMLET upon OA binding (when compared with EL-OA) may be associated with the enzymatic properties that impart diseased cell specificity and encapsulation propensity. It has been proposed that the targeting capabilities of the enzyme complex stem from the protein moiety (Casbarra et al. 2004; Brinkmann et al. 2013) of the group. In this interpretation, it is the “dynamics of the protein” in the ligand–protein complex that alters the activity of the lipid (OA) and simultaneously modifies the sensitivity of the targeted membrane to ion transport inhibition. This suggests that the protein has a more active role than simply delivering the OA molecule to targeted cells but rather has an integral role in adapting specific structural and functional characteristics in the ligand-bound protein that may vary depending on the protein involved in the actual complex.

EL-OA has been found to be toxic to both healthy and nonhealthy mammalian cells, whereas HAMLET is constrained to act only on cancerous cells. HAMLET has also been found to possess moderate bactericidal activity toward certain bacterial pathogens that target the respiratory tract (Clementi et al. 2013). In this capacity, its activity toward bacteria closely mimics the mechanism utilized in eukaryotic cell apoptosis. Particularly, once HAMLET is adsorbed onto the directed membrane it is internalized through endocytosis and accumulates inside of the nucleus of the targeted cell (Johnke and Petersen). This internalization process eventually leads to membrane degradation by causing membrane permeability, which is subsequently followed by an (preexisting) apoptosis promoting P38 pathway. Unlike EL-OA that acts directly on target membranes, HAMLET acts through interaction with intracellular targets that are not accessible in healthy cells. Hence, the general mechanism for association with membranes in both bacterial and eukaryotic cells is far more intricate than when contrasted with EL-OA (supplementary material S3, figs. S3 and S4, Supplementary Material online).

We conjecture that the differences in the functionality of EL-OA when contrasted with HAMLET may be attributed to distinctive intraprotein interactions in the two systems that are excited upon OA binding. Principally, we find that a conglomerate of extended surface fluctuations (figs. 2*b*, 2*d*, and 4*b*) distinguish HAMLET from EL-OA and we postulate that these attained attributes refine the cytotoxic activities of the former enzyme complex. This hypothesis is further supported in our experimental measurements, where we also detect a more extensive network of surface fluctuations that permeate globally within the protein structure in HAMLET (fig. 1*d* and supplementary fig. S4, Supplementary Material online) when contrasted with that of EL-OA.

##### Computational Analysis of Correlated Fluctuations and Dynamics of Coevolutionary Links in Conventional Lysozyme (lysozyme) Complexed with the Inhibitor (NAG)_3_.

Computational measurements of lysozyme highlight the principal, dynamical motions that are induced in response to ligand-binding (fig. 2*e* and supplementary fig. S7*e*, Supplementary Material online). At first glance, one of the most conspicuous distinctions that we uncover in the analysis of lysozyme dynamics (when contrasted with the antimicrobial properties of the two other proteins that we have considered in this work) is a shift away from the dominance of the A/B loop (specifically Asn 19 and Tyr 20) in the protein functional motions. In our analysis of c-type lysozyme dynamics, the A/B loop motion appears to be linked with modulating macromolecular-binding affinity/interactions on the protein surface. Hence, the shift away from the A/B loop as a focal feature in the governing protein fluctuations may somehow be interrelated with more sophisticated approaches for ligand-binding and specificity.

From the computational analysis of lysozyme dynamics when bound to the ligand (NAG)_3_, we determine that Val 109 is central to the development of long-range coherent interactions in the protein. It functions by forming a bridge between neighboring residue clusters through both H-bonds and electrostatic interactions as well as by extending long-range contacts to other (coevolutionary) communities that influence hydrophobic packing interactions that connect flexible loops and strands that form and support catalysis in the protein active-site (fig. 4). Analogous to what we observed in the HAMLET complex, we again identify a connection between Val 109 and Arg 61(fig. 4*a* and *b*) in lysozyme that forms hydrophobic contacts between the ligand and the protein. The protein–ligand coupling creates a dynamic link that is extended to β-sheet residues in addition to residues establishing the aromatic cluster I. As a result, the network of interactions initially formed in the hydrophobic protein core is transmitted as torsional fluctuations that are predominantly concentrated on Helix D, the 3_10_-helix in the α-domain and a limited portion of the A/B loop (fig. 2*e*).

We also identify functional links involving Gly102 with Val 109 in the lysozyme–(NAG)_3_ network (fig. 4) where the interaction is associated with the excitation of residues involved in substrate positioning. Gly 102 has an intermediate MI value in the coevolution network and is central in connecting two distinct network communities (fig. 4). In addition to its connection with Val 109, Gly 102 is strongly correlated with the high MI residue Leu 75. Hence, Gly102 connects residues in the solvent accessible loop with residues in the long-loop region of the protein structure. The modification in coevolutionary links that are activated with lysozyme ligand-binding, and that differ considerably from both EL-OA and HAMLET, ultimately unites water-mediated interactions on the protein exterior with hydrophobic interactions in the protein core. The intersection of the two communities is responsible for creating a hinge point about Gly 102 that separates the juncture of the α- and β-domain that actively plays a role in positioning the ligand into the protein active-site.

Also unique to lysozyme is the increase in intramolecular and intermolecular H-bonds in response to ligand-binding. Further, there are additional weak mechanical forces introduced by the ligand–protein interactions in the core that are strongest on the positioning residues lining the active-site, specifically Phe 34, Asp 52, Asn 59, Trp 62, Leu 75, Gly102, and Asn103 (supplementary material S7, fig. S7*e*, Supplementary Material online). The internal strain created by the closely knit network of inter- and intramolecular protein interactions is distributed and propagated by a twisting motion about the hinge point separating the two domains. The hinge bending motion is associated with refining and optimizing the affinity and recognition of the ligand (Ma et al. 2002), comparable to what has been observed in HAMLET, but on a more comprehensive level. Particularly in this case, we observe a progression of antimicrobial properties that are brought about by increasing specificity for the target macromolecule predominantly through specific intramolecular and intermolecular H-bonding interactions.

**Fig. 5.**
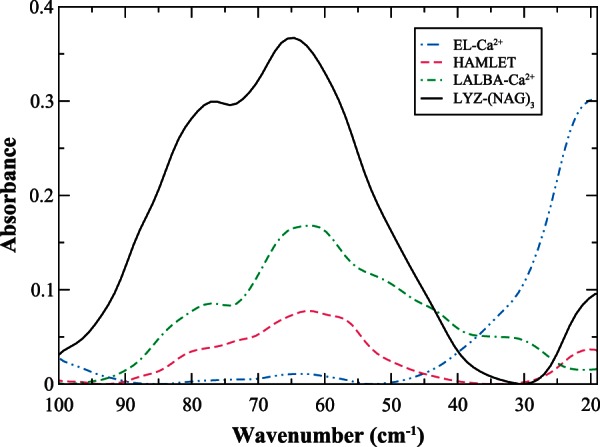
Experimental THz spectrum of EL-Ca^2+^ (blue, dotted-dashed line), HAMLET (magenta, dashed line), LALBA-Ca^2+^ (green, dotted-dashed-dotted line), and hen egg-white lysozyme (LYZ)-(NAG)_3_ (black, solid line) in the 20–100 cm^−1^ spectral region at 300 K.

It has been stated that the function of a protein may change across an evolutionary time scale leading to a modification in the propensity of the (sub)states within the ensemble due to constraints on the individual amino acids (Romero and Arnold 2009). The alteration of the possible protein conformations can rapidly reshape the overall energy landscape and hence, drastically alter the functional motions of an individual protein in a protein family. Previous work (Woods 2014b) on lysozyme has demonstrated that the global structural fluctuations are considerably restrained when bound by the ligand (NAG)_3_ in comparison with the apo-state protein dynamics. Ligand-binding promotes a collective torsion that dynamically reorganizes the weak interactions of residues both adjacent and distant to the active-site that bias the dynamics of the protein toward a subset of conformations that favor a more rigid protein functional state (fig. 5 and supplementary material S4, Supplementary Material online). In the ligand-bound state, the global interaction network involving weak interactions in the THz regime plays a direct role in modulating substrate-binding affinity in the enzyme binding pocket. Hydrogen bonding between the protein and its ligand as well as to solvent molecules on the protein surface function together to provide a directionality and specificity of interaction that is a fundamental aspect of lysozyme molecular recognition (Xu et al. 1997). The energetics and kinetics of hydrogen-bonding are therefore optimized for rapid sampling of a narrower conformational space, facilitating stability to the protein structure and providing the specificity required for attaining highly selective macromolecular interactions.

##### Summary: Shifts in Coevolutionary Networks and the Antimicrobial Properties of EL-OA, HAMLET, and Lysozyme–(NAG)_3_.

Interestingly, with the exception of conventional lysozyme (LYZ gene), all of the proteins in the c-type lysozyme subfamily are partially unfolded in the nonliganded state and nonexclusive ligand-binding both stabilizes and imparts diverse conformational and functional effects in the individual proteins. An interesting viewpoint of protein evolution embraces the position that novel protein function ascends from precursor proteins that are conformationally heterogeneous (Zimmermann et al. 2006; Hou et al. 2007). From this perspective, the c-type lysozyme MG state represents a highly dynamic conformational ensemble. The structural heterogeneity associated with the state as well as other stable, functional intermediates has been proposed as a viable and essential means for promoting evolutionary adaptability within protein and enzyme families (Romero and Arnold 2009; Tokuriki and Tawfik 2009; Nagulapalli et al. 2012). For example, genomic and structural data have indicated that the proteins in the c-type lysozyme subfamily are related by a common ancestor (Prager and Wilson 1988; Meier and Ozbek 2007; Irwin et al. 2011). It is presumed that through both mutation and evolutionary divergence that a “shift in coevolutionary relationships” has advanced the individual functional properties of the enzymes within the family. In previous sections we observed that EL and LALBA share many common structural similarities and bind a mutual ligand, yet retain very different functional properties. In the native state of both proteins, the origin of the variance in function is linked with active-site sequence variations and a subsequent modification in interdomain coupling. In this study, we have assumed that LALBA evolved from calcium-binding lysozyme. In fact, it has been conjectured that the duplication creating the lactalbumin and calcium-binding lysozyme genes predated the bird–mammal divergence (Grobler, Rao, et al. 1994). In both proteins, the MG state provides an equilibrium between several different conformations that facilitates binding of more than one ligand. Mutation would likely shift the equilibrium of the conformational ensemble. Therefore, over the evolutionary time scale selected mutations might be expected to increase the affinity and/or refine the selectivity associated with recognition or binding of the targeted substrate of the specific proteins by adjusting the population of accessible conformations (Zimmermann et al. 2006). To some extent, this was demonstrated through binding of OA to both EL and LALBA. The HAMLET complex was found to possess more refined cytotoxic activities when contrasted with EL-OA. This was attributed to the formation of long-range intraprotein interactions and hence, alteration in propagation pathways (linked with conformational fluctuations) associated with enhanced affinity and specificity on the protein surface. In this interpretation of evolutionary adaptability, one might deduce that conformational restriction is a natural accompaniment of evolutionary progress (Zimmermann et al. 2006). Incidentally, unlike LALBA and EL, lysozyme does not possess an MG state nor has it been shown to have any known cytotoxic activities. However, lysozyme has acquired refined antimicrobial properties that arise in response to a shift in interactions that ultimately promote a progression toward greater ligand–protein specificity. In this view, lysozyme represents a turning point in the evolutionary divergence of the antimicrobial properties of the structurally similar proteins in the family. Incidentally, the role of the enzyme is to break down peptidoglycans found in bacterial cell walls.

### Functional Diversity Evolving from Limited Sequence Diversity in the c-Type Lysozyme Subfamily

**Fig. 6.**
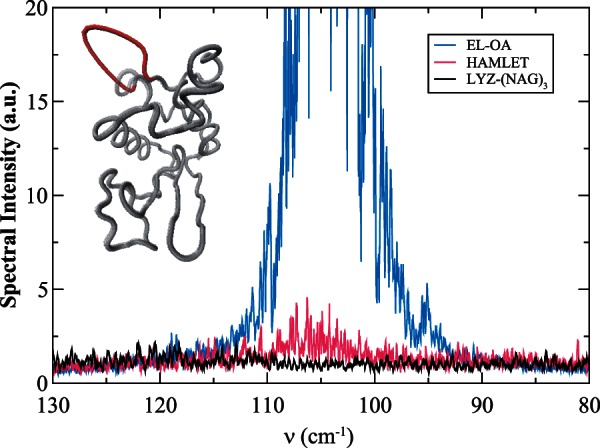
Fourier transform of the VACF of residues comprising the A–B loop in EL-OA (blue), HAMLET (magenta), and LYZ-(NAG)_3_ (black) from the MD simulations in the 80–130 cm^−1^ spectral region. The figure in the inset depicts the motion of the A–B loop in red overlaid on a gray, C-α tube representation of the structure of EL.

Moving from one function to another in a protein family requires the correlated evolution of key amino acid residues. These functionally coupled residues have been found to function as facilitators that promote activities such as protein–macromolecule associations and/or contribute to enzymatic catalysis (Liu and Bahar 2012). It has also been proposed that these particular types of local, structural fluctuations may also provide the basis for functional diversity in a protein family and shape the route to evolutionary divergence of new functions (Daily and Gray 2007; Glembo et al. 2012). In four of the protein–ligand systems that we have explored in this work, we have deduced that localized fluctuations of a particular flexible loop (the A/B loop) are connected with creating macromolecular affinity subsites on the protein surface. In the c-type lysozyme sequences that we have studied thus far, the flexible loop in the protein family contains coevolved residue pairs that are in close spatial proximity (fig. 3*c*). When mutations occur within this network of close proximity residues (with high MI values) that comprise the loop region, the localized source of flexibility that the loop imparts becomes disrupted and central protein interactions within the core (that are interconnected with the loop motion) are noticeably altered, as is the overall function of the protein. In particular, the sequence comprising the A/B loop in both EL and LALBA has a particular high MI residue, Gly 19, which is pivotal in communicating the localized flexibility in the loop region with function defining hydrophobic networks in the protein interior (fig. 4*a*–*c*). The corresponding residue in lysozyme is mutated to an asparagine. The selected mutation has a profound effect on both the dynamics and geometry of the loop when compared with the other protein–ligand systems considered in this study. Particularly, the exchange of glycine for asparagine in a key position in the arrangement of the loop results in a relatively rigid A/B loop (fig. 6) when compared with either LALBA or EL and a significant alteration in the loop protein intramolecular contacts that regulate the flexibility of other helical regions in the α-domain. Specifically, the mutation of Gly for Asn in a central portion of the loop leads to weakened interactions between hydrophobic core residues Trp 111 and Trp 108 when Gly 19 is replaced with Asn (supplementary material S5, fig. S5, Supplementary Material online). Moreover, the adaption in residue packing in the protein interior is also tied with modified dynamics in Helix D and parts of the 3_10_-helix in the α-domain. The residue exchange in the flexible loop region is interconnected with the generation of novel, intramolecular H-bonds that initiate molecular changes in the protein substrate-binding pocket.

The combined modifications that arise with the mutation ultimately mediate structural rearrangements that arbitrate collective motions between the domains (hinge-bending motion) (fig. 5 and supplementary material S4, Supplementary Material online). Hence, this shift in hydrophobic residue interactions and the subsequent structural readjustments that take place in the 3D protein structure are decisive for high-selectivity ligand-binding. The disruption of intramolecular contacts and the consequent alteration of dynamics of α-domain helical regions increase the free energy barrier heights between selected conformers, effectively restricting the window of accessible substates in the dynamic ensemble. In brief, the attenuation of A/B loop dynamics in lysozyme is directly linked with the propensity of sampling different structural isomers of similar energy. Specifically, the mutation at a spatially distant site exerts conformational rearrangements within the protein that lead to a shift in the distribution of populations that strongly influence the molecular shape of the binding cavity and simultaneously constrain the range of conformations to those that favor high-affinity ligand-binding. These seemingly minor adjustments in the surface, flexible loop dynamics are fundamental in determining two very distinct properties of lysozyme that are unique when contrasted with the other structurally similar proteins in the superfamily: 1) Lysozyme has no MG state and (2) lysozyme only binds a single type of ligand. In this case, conformational restriction appears to motivate protein evolution. The replacement of a highly fluctuating loop with a rigid structural unit brings about the stabilization of the protein structure and simultaneously advances the affinity and functional selectivity that refines the protein’s recognition mechanism to a substrate. In summary, the connected surface sites formed by the motion of the A/B loop in the c-type lysozyme subfamily appear to be an evolutionarily conserved dynamical network that constructs preferred sites for the emergence of allosteric control on the protein surface. When perturbations are introduced to these specific surface positions, they appear to have the ability to initiate conformational adaption over protein function.

### Detection of Experimental Interaction Networks

#### Ensemble View of Proteins and the Experimental Detection of Allosteric Communication Pathways

The ensemble view of proteins can contribute mechanistic insight into the evolution of their enzymatic functions. Additionally, this view can also be used to highlight the close relationship between conformation and dynamics in proteins that is required for developing a molecular level understanding of allostery. It is generally accepted that allosteric propagation takes place through dynamic fluctuations in protein intramolecular contacts (Daily and Gray 2007; Nussinov 2012).

Experimentally, we are able to detect more localized interactions taking place in the protein structures studied. These networks of interactions detected generally comprise a subset of the molecule rather than the entire protein and have vibrational frequencies in the 100–250 cm^−1^ spectral region. The motions detected in this higher frequency part of the THz spectrum are sensitive to local relaxations that reflect specific intramolecular and intermolecular thermally induced fluctuations that are driven by external perturbations in the individual protein systems (Woods 2014c). Moreover, these localized intermolecular interactions have been hypothesized to form the basis of allosteric signal propagation in proteins (Liu and Bahar 2012). Consequently, THz spectroscopy not only has the potential to provide insight about the fast, localized fluctuations that are correlated with protein flexibility but also affords valuable information about the interdependence between global, collective motions and the localized interactions that act as preliminary determinants in allosteric signal transmission.

**Fig. 7.**
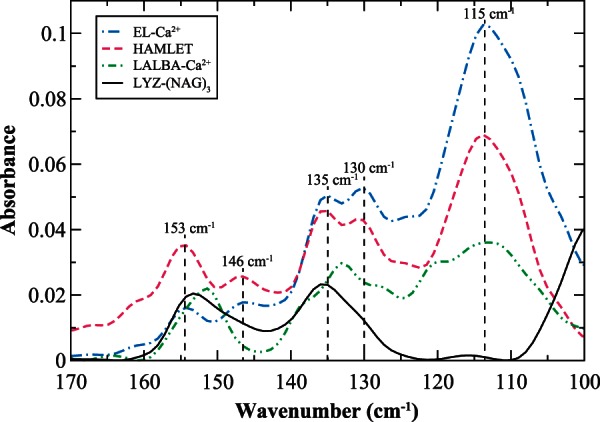
Experimental THz spectrum of EL-Ca^2+^ (blue, dotted-dashed line), HAMLET (magenta, dashed line), LALBA-Ca^2+^ (green, dotted-dashed-dotted line), and hen egg-white lysozyme (LYZ)-(NAG)_3_ (black, solid line) in the 100–170 cm^−1^ spectral region at 300 K.

In figure 7, an experimental assessment of the localized network of interactions taking place in four of the different protein–ligand systems previously discussed in this investigation, in the 100–170 cm^−^^1^ spectral region, uncovers that the dynamics of binding is indeed distinct for each of the samples considered. For example, the peak at approximately 115 cm^−^^1^ in figure 7 is dominated by the torsional oscillation of the A/B loop in the protein–ligand systems investigated. In the El-Ca^2+^ spectrum the 115 cm^−^^1^ mode is most prominent suggesting that the localized fluctuation of the loop dominates the network of intermolecular interactions, whereas in the lysozyme–(NAG)_3_ sample the same mode is greatly reduced implying a diminished role in the functional dynamics of the ligand–protein system. The experimental results are in line with the computational results of the analogous protein–ligand systems that we have already encountered in this study. A closer inspection of the THz experimental spectra also reveals that there are other modes visible that may help to elucidate the role of these detected relative instabilities in the protein–ligand network of interactions that have been proposed as a means to modulate or shift substrate-binding affinity.

#### Effect of the Solvent on the Dynamic Distribution of Conformational Substates and the Evolution of Ligand-Binding Affinity and Specificity in c-Type Lysozymes

In the experimental detection of the protein interaction networks in figure 7, we find that EL-Ca^2+^ and HAMLET both contain heterogeneous water-mediated interactions that may be linked with the mechanism of adjusting the equilibria of the ensembles in the protein to favor specific ligand-binding interaction or to aid in molecular recognition (fig. 5 and supplementary material S6, Supplementary Material online). In addition to the prominent 115 cm^−^^1^ mode that has been attributed to A/B loop dynamics, we identify the approximately 146 cm^−^^1^ protein–solvent coupled mode in both samples that is linked with water molecules arranged in a highly tetrahedral molecular arrangement and additionally another peak at 153 cm^−^^1^ that is ascribed to protein–solvent H-bonding interactions that take place with water molecules arranged in an unstructured (distorted H-bond) molecular conformation (Woods 2014a, 2014b). The interplay between the distinct local structures of the solvent molecules in the hydration shell and their H-bond dynamics with the protein is conceivably linked with relaxation-induced reorganizational processes that alter conformer barrier heights and accordingly, also shift ligand-binding affinities.

In both EL-Ca^2+^ and HAMLET, the presence of heterogeneous protein–water interactions appears to be directly related to the formation of localized instabilities in the protein structure. For instance, the 130 cm^−^^1^ mode in figure 7 is strikingly apparent in the spectra of both protein–ligand systems, suggesting that solvent has a strong influence in modulating the localized motions that impact the dynamics on the surfaces of these specific proteins. This observation is in line with our results from MD simulation in earlier sections that have indicated that the local structural fluctuations in both EL-Ca^2+^ and HAMLET are connected with a network of intraprotein interactions that are coupled with protein core defects that produce and ensemble of states that are optimized for macromolecular recognition and affinity on the protein exterior. Further, we postulate that the diverse solvent-mediated protein interactions detected in the experimental networks of EL-Ca^2+^ and HAMLET play a central role in optimizing short-range intraprotein communication as well as stabilizing localized side-chain fluctuations in both proteins. Both of the ligand-bound proteins are found to possess very limited interdomain coupling but exhibit large-scale A/B loop dynamics; this is particularly apparent in the experimentally detected global and local structural fluctuations in EL in figures 1 and 5. In our computational assessment of EL-Ca^2+^ and HAMLET, we find that this local structural instability is likely used as a fine-tuning mechanism for regulating the features of macromolecular recognition and binding specificity on the protein surface.

The combined experimental and computational results suggest that the ligand sensitive equilibrium in ancestral c-type lysozymes can be adjusted by the evolution of site-specific local instabilities within an individual protein. Moreover, solvent-induced interactions appear to be active components in allosterically shaping the energy landscape that dictates collective motion and therefore, binding selectivity. For example, in contrast to EL-Ca^2+^ and HAMLET, the 130 cm^−^^1^ mode in figure 7 is greatly reduced or nearly absent in the experimental spectrum of LALBA-Ca^2+^ and lysozyme–(NAG)_3_, respectively. In LALBA and lysozyme, interdomain coupling and long-range intraprotein communication are maximized through ligand-induced modifications in intra- and intermolecular hydrogen-bonding networks. The result is a succession of correlated, conformational rearrangements in the protein structures through which specificity for their respective substrates is amplified. Additionally, in the 100–170 cm^−^^1^ experimental spectral region, we only detect a single protein–solvent coupled translational mode at 153 cm^−^^1^ in both lysozyme–(NAG)_3_ and LALBA. In previous investigations, the sole presence of the 153 cm^−^^1^ mode in the protein–ligand solvent-coupled translational region has been attributed to the development of a high degree of coupling between protein local structural fluctuations, ligand-binding, and protein global conformational changes (Woods 2014a, 2014b). Its presence has been directly correlated with the formation of large-amplitude anharmonic motions that activate long-distance communication among residues that are distantly located in the protein 3D structure. MD simulation and correlational analysis of the native state of LALBA and ligand-bound lysozyme have indicated that there is a significant loss of flexibility in key residues, both adjacent and distant from the respective ligand-binding sites that accompany the progression of collective motion in the proteins that we surmise is fundamental to functional divergence in the evolutionary path of c-type lysozyme proteins. The modification in molecular interactions reflects a variation in the sampling of conformational ensembles and concurrently the stimulus for the acquisition of novel functions. We find that solvent-coupled protein fluctuations have a decisive influence on the conformational rearrangements that take place within the individual proteins and consequently, the distribution of conformers that form the underlying gradient of binding-site conformations that shape the evolutionary foundation for ligand-binding affinity and recognition in the structurally similar proteins.

## Conclusions

In this investigation, we compare three proteins that serve as representative structures in the c-type lysozyme subfamily. We have employed both experimental and computational methods to uncover allosteric interactions that are believed to play a central role in determining the characteristics of the specific molecular networks that ultimately determine protein function. Toward this endeavor we have utilized MSA methods to map out the globally encoded architecture of amino acid interactions in the protein family and analyzed the probable network of connected residues that link distant functional sites in the protein tertiary structure. In addition, we have experimentally probed the intrinsic dynamics of individual proteins within the protein family that direct the selection and sampling of the conformational substates and directly probed the intra- and interprotein dynamics that underlie their allosteric propagation pathways.

The structurally similar proteins investigated occupy a common conformational ensemble (Keskin et al. 2000; Maguid et al. 2005) with the conformational states of the individual proteins populated to different extents. Mutations stabilize and optimize a subset of conformations that is optimal for the specific protein primary function. This is particularly evident when considering the antimicrobial properties of distinct proteins in the family. In this context, we observe an “evolution” of antimicrobial attributes that are achieved by increasing specificity for a target substrate through specific intramolecular and intermolecular H-bonding interactions and that simultaneously prompt shifts in the equilibrium of the ensemble of conformational states. In this case, the sequence diversity in the protein structures is presumably driven by selective advantages tailored toward microbial defense or adaptation. Conformationally heterogeneous protein structures ensure recognition of a broad range of target molecules, while recognition of a particular foreign molecule favors mutations that increase the affinity and selectivity associated with molecular recognition.

The proteins comprising the c-type lysozyme subfamily are relevant examples of biological systems in which allosteric modulation plays a central role in their function and where biological divergence through selective pressures on ensembles has distinctly resulted in the acquisition of novel functions. Even recently, it has become apparent that a molecular level understanding of the targeting mechanism of members within this protein family could be instrumental in the development of nontoxic antitumor drug targets (Svanborg et al. 2003; Mercer et al. 2011; Ho CS et al. 2012) as well as in the rational design of antimicrobial agents that are effective against bacterial strains that have become resistant against conventional antibiotics (Clementi et al. 2013; Marks et al. 2013).

## Materials and Methods

### THz Spectroscopy Experiments

The THz spectroscopy experiments were carried out on a Jasco FTIR—6000 series spectrometer. The hydrated protein film samples were collected with a liquid helium cooled bolometer in the 15–250 cm^−1^ spectral range. The 15–100 cm^−1^ THz spectra were collected with a 25 -µm beam splitter, whereas the data in the 100–250 cm^−1^ spectral region were collected with a 12 -µm beam splitter. For each transmission measurement, a 25-mm-diameter region of the protein sample was illuminated with the THz beam to determine the absorbance. In the spectral measurements presented, each scan consists of 16 averaged scans and the infrared data were collected with a spectral resolution of 4 cm^−1^. The temperature of the samples was varied using a SPECAC variable temperature cell with an adjustment range of −190 to 30 °C.

In our analysis of the experimental data, we operate under the assumption that THz spectroscopy detects fluctuations of dominant conformations within an ensemble. This is particularly the case when considering the dynamics in protein–ligand systems. Additionally, a conformational selection model (Frauenfelder et al. 1991) is adopted in the description of protein dynamics in the absence of or leading up to ligand-binding and the induced fit model (Koshland 1958) is utilized in the description of the subsequent optimization of interactions that occurs after ligand-binding has taken place (Boehr et al. 2009).

### Sample Preparation

#### EL Complexed with Ca^2+^ (EL-Ca^2+^)

EL was extracted from horse milk powder that was purchased from De Lage Wierde (Wirdum, The Netherlands). The milk was dissolved in distilled water at a concentration of 2.5 mg/ml and left overnight at 4** °**C. The following day the milk solution was defatted by spinning at 5,000 g for 10 min. The resulting skim milk was further diluted in a buffer of 20 mM Tris–HCl, pH 8.0 and run through a Pierce cation exchange column. The lysozyme and LALBA components were eluted from the skim milk with a buffer containing 1 M NaCl. Desalting columns were used to remove excess salt from the eluted proteins and hydrophobic interaction chromatography was used (Noppe et al. 1999) to separate LALBA from lysozyme by eluting lysozyme with a buffer including 10 mM Ca^2+^. The native state of lysozyme was confirmed with near-UV CD spectroscopy and the purity by sodium dodecyl sulfate polyacrylamide gel electrophoresis. The apo-EL state was prepared by ethylenediaminetetraacetic acid (EDTA) treatment (Svensson et al. 2000) of the purified native state by adding 3.5 mM EDTA to remove bound Ca^2+^.

#### LALBA Complexed with Ca^2+^

Human LALBA was purchased from Sigma-Aldrich (St Louis, MO). The lyophilized powder was first dissolved in a buffer of 20 mM Tris–HCl, pH 8.0 and placed in desalting spin column to remove excess salt. The desalted pellet was resuspended in a buffer of 20 mM Tris–HCl, pH 8.0. UV absorbance was used to determine the sample concentration, which was subsequently diluted to a concentration of 1 mg/ml. 3.5 mM EDTA was added to an aliquot of the stock sample to create the apo-protein, whereas 10 mM CaCl_2_ was added to another portion to restore the native state. Both the native state and the apo-state of α-lactalbumin were verified with near-UV CD spectroscopy and (mid-) infrared spectroscopy.

#### HAMLET and EL-OA

Apo- state proteins were interacted with anion exchange columns (Svensson et al. 2000; Svanborg et al. 2003; Brinkmann et al. 2013) that had previously been preconditioned with OA. HAMLET and EL-OA complexes were formed by adding apo-EL or apo-LALBA to the preconditioned columns in a 1:1 molar ratio in a buffer consisting of 20 mM Tris–HCl, pH 8.0 followed by elution with 1 M NaCl. The eluted complexes were desalted by dialysis against water, lyophilized, and again resuspended in a buffer containing 20 mM Tris–HCl, pH 8.0. The final concentration of the samples was determined by UV absorbance.

#### Hen Egg White Lysozyme–(NAG)_3_

HEWL was purchased from Sigma-Aldrich. To remove excess salt and other particles from the sample prior to the experiment, the lysozyme sample was dissolved in water and run through a desalting spin column with a buffer consisting of 10 mM NaH_2_PO_4_ and 0.01 mM EDTA at pH 7.0. The lysozyme sample used in the experiments was initially prepared by diluting a stock solution to a concentration of 1 mg/ml. The (NAG)_3_ was also purchased from Sigma and the HEWL-(NAG)_3_ samples were prepared by adding a slight excess of the substrate relative to the diluted protein in solution. Reaction volumes of 1 ml of the HEWL-(NAG_)3_ solution were created and placed in a 4°C refrigerator overnight and prepared the following day for future experiment.

#### Protein Film Samples Used in the THz Spectroscopy Experiments

All of the protein and protein–ligand samples were prepared as unoriented films for experiment. The protein film samples that were used in the THz spectroscopy experiments were prepared by allotting 20 µl of the prepared sample onto a high-resistivity silicon window. Excess water from the solution was initially removed by applying a low, steady flow of N_2_ gas over the sample droplet for approximately 10 min. The resulting protein film was rehydrated by equilibrating the partially dried sample in a vacuum sealed container with the vapor pressure of a saturated salt solution at 20 °C for a minimum of 5 days. A relative humidity of 97% was obtained from the vapor pressure of a saturated K_2_SO_4_ solution (Wexler and Hasegawa 1954). Previous experimental measurements (Woods 2010, 2014b) on c-type lysozyme proteins have found that at this hydration level, the water molecules available in the hydration layer are sufficient for completing both the first and second hydration shell of the protein (Sartor et al. 1995). The prepared film samples were placed in a sealed transmission cell consisting of two high resistivity silicon substrates and a saturated salt solution was placed at the bottom of the cell to ensure that hydration was maintained throughout the experiment.

### MD Simulations

#### Equilibrium MD Simulations

The MD simulations were carried with the Gromacs package (www.gromacs.org) version 4.6 using the GROMOS96 43a2 force field parameters (van Gunsteren et al. 1996; Pronk et al. 2013).

##### Calcium-Binding Protein Simulations (El-Ca^2+^ and LALBA-Ca^2+^).

The starting structure of EL was initially downloaded from the protein databank (2EQL.pdb). In the EL simulations, 2,778 water molecules were added, in which the SPC model of water was used and a calcium ion was placed in the known calcium-binding site. Protein data bank (PDB) structure 1A4V.pdb was used as an initial preliminary structure for the MD simulations carried out on human LALBA. In the calcium-bound LALBA simulations, 2320 SPC model of water molecules were added to the sample box. In both (EL and LALBA) calcium-bound protein simulations, energy minimizations were carried out to reduce the number of unfavorable contacts between the initially added solvent molecules and the protein using a steepest descent method to a convergence tolerance of 0.001 kJ mol^−^^1^. The energy minimization was followed by an MD run with constraints for 200 ps in which an isotropic force constant of 100 kJ mol^−^^1^ nm^−^^1^ was used on the protein atoms. During the restrained dynamics simulation, the temperature and pressure of the system were kept constant by weak coupling to a modified velocity rescaled Berendsen temperature (Berendsen et al. 1984) and pressure baths and in all cases the protein, water, and calcium ion have been coupled to the temperature and pressure baths separately. The final output conformation from the MD simulation with constraints was used as the starting conformation for an initial 50 ns equilibrium MD simulation. Ten subsequent simulations were conducted where conformations from the last 10 ns of the equilibrium simulations were used as starting point conformations for each different simulation. These succeeding simulations were carried out for an additional 50 ns and were eventually used to assess the picosecond time scale fluctuations in the protein systems. The final simulations were carried out with a 1-fs time step where the bonds between the hydrogen and the other heavier atoms were restrained to their equilibrium values with the linear constraints (LINCS) algorithm (Hess et al. 1997). Particle mesh Ewald method (Essmann et al. 1995) was used to calculate the electrostatic interactions in the simulation and was used with a real-space cutoff of 1.0 nm, a fourth order B-spline interpolation, and a minimum grid spacing of 0.12 nm.

##### MD Simulations of the MG State of EL and LALBA and the Formation of HAMLET and EL-OA Complexes.

The structures obtained from the native state simulations were used as starting points for the partially unfolded states of LALBA and EL. In each case, the calcium ion was removed from the binding pocket of the native state structures and heated slowly for 1 ns to 368 K. After the initial heating at 368 K a subsequent run was initiated for an additional 10 ns to equilibrate the system and the progress of unfolding was monitored by measuring the radius of gyration and comparing it with the native state protein. A random selection of partially unfolded structures was selected from the last 5 ns of the heated, equilibration runs and used for the formation of HAMLET and EL-OA complexes. In each protein–OA simulation, a single OA molecule was placed in the simulation box containing the partially unfolded protein and solvent molecules. The OA GROMACS topology was initially built from the PRODRG server (Schüttelkopf and van Aalten 2004) and the charges further refined by using the quantum chemistry software program ORCA (https://orcaforum.cec.mpg.de/). Initial energy minimization runs of the complexes were carried out and followed by an MD run with constraints as outlined in the previous section. Equilibrium runs similar to those carried out for the native protein at 300 K consisted of one protein molecule, one OA molecule, and solvent molecules. The equilibrium runs were carried out for an additional 20 ns in an effort to determine the most favorable position of the OA molecule in the protein complex. In all, out of the 25–20 ns equilibrium simulations carried out for each complex there were clear indications that there was a preferable, stable binding position of the single OA molecule in the different protein molecules. Monitoring the stability of the position of the OA during the 20-ns simulation was the manner in which the stability was assessed. In the HAMLET simulation, there was a single stable position of the OA molecule and this was when the fatty acid molecule was weakly bound by electrostatic interactions in the LALBA active-site with the carboxylic head pointing outward from the cleft. All other positions of binding resulted in transient mobility of the OA molecule after at most a few nanoseconds of binding. Similarly, in the preferred binding conformation of OA in the EL-OA complexes, the OA molecule was found to be stable when weakly bound to the protein by residues residing at edge of the long-loop of the protein. The stable conformations of EL-OA and HAMLET were used for the final production runs with parameters analogous to those outlined for the native state proteins. The final simulations were conducted for 50 ns and the last 10 ns of the simulations was used for analyzing the picosecond time-scale fluctuations of the protein and the ligand. It is important to note that experimental investigations on both HAMLET and EL-OA complexes have indicated a much higher OA:protein ratio (Marks et al. 2012; Brinkmann et al. 2013; Clementi et al. 2013) than we have used in our MD investigation. The reason that we have focused our simulation efforts with a 1:1 ratio of OA to protein is that when trying to introduce more than a single OA molecule to the simulation box, the OA molecules consistently formed micelle structures rather than interacting with the protein.

##### Molecular Docking of EL-OA and HAMLET Complexes.

SwissDock (http://swissdock.vital-it.ch/) web services (Grosdidier et al. 2011a, 2011b) were used as a secondary verification to determine the binding sites of OA in LALBA and EL. We assumed a blind docking estimate of the binding modes comprising the most favorable energies in the docking calculations. For the most part, the results were in line with the outcome of our MD simulation studies on both complexes. The highest populated cluster in HAMLET predicted a conformation with the OA molecule in the LALBA active-site cleft with the carboxylic head facing outward toward the solvent interface, whereas the lowest energy cluster favored a ligand orientation close to the long-loop residues with binding specifically to residues 68–70. In EL-OA, the highest populated cluster consisted of a conformation where the ligand binds in the protein active-site. The lowest energy cluster of EL-OA featured the ligand bound near the C-terminal helix of the protein. The output of the docking results was visualized with the UCSF Chimera (http://www.cgl.ucsf.edu/chimera/) molecular modeling system (Pettersen et al. 2004).

#### Calculations

##### Velocity Autocorrelation Function.

The velocity autocorrelation function (VACF) of atoms from the MD simulations was computed with the extended analysis tools that are included as part of the Gromacs software package. The VACF is defined by

(1) $$C_{\nu }\left(\tau \right)=\frac{\langle v_{i}\left(\tau \right)\cdot v_{i}\left(0\right)\rangle }{\langle v_{i}{\left(0\right)}^{2}\rangle },$$

where *v* refers to velocity and *i* denotes an atom or molecule in the simulation system. Fourier transform of the VACF (VACFFT) is used to project out the underlying frequencies of the molecular processes associated with the correlated motions detected in the simulation (Woods 2010; Ding et al. 2011; He et al. 2011).

##### Principal Component Analysis and Full Correlation Analysis.

Principal component analyses (PCAs) were carried out by diagonalizing the covariance matrix $C_{ij=\langle \left(x_{i}-\langle x_{i}\rangle \right)\left(x_{j}-\langle x_{j}\rangle \right)\rangle }$, where *x* denotes protein atomic positions in the 3*N*-dimensional conformational space and the angular brackets represent the averages over the MD trajectory. Translational and rotational motions were removed by a least squares fitting to a reference structure. The eigenvectors of ***C*** were determined by diagonalization with an orthonormal transformation matrix. The resulting eigenvectors from the transformation were used to determine the PCA modes with eigenvalues (λ) equivalent to the variance in the direction of the corresponding eigenvector. The MD trajectory was projected onto the principal modes to determine the principal components. The eigenvalues $\lambda_{i}$ of the principal components denote the mean square fluctuation of the principal component *i* and are arranged so that $\lambda_{1}\geq \lambda_{2}\geq ...\geq \lambda_{3N}$. Using this arrangement, the trajectories were filtered along the first principal component to analyze the collective dynamics taking place within the protein. The cosine content of the PCA modes presented was less than 0.005.

Full correlation analyses (FCA) on the MD trajectories were carried out by using the algorithm developed by Lange and Grubmüller (2006). FCA uses MI to identify correlations of residues from the MD trajectory. Fluctuations in the coordinates of the protein are considered to be uncorrelated if

(2) $$p(x)=\prod_{i=1}^{3N}p_{i}(x_{i}),$$

where ***p***(***x***) signifies the canonical ensemble given by $p(x)=\frac{e^{-E_{i}\beta }}{Z}$, in which Z refers to the partition function, *E* the energy, β the inverse temperature, and $p_{i}(x_{i})=\int p(x)dx_{i\neq j}$ the marginal density. If there are correlations in the system then they can be identified by MI,

(3) $$I(x_{1},x_{2},x_{3},\ldots x_{3}{}_{N})=\sum_{i=1}^{3N}H(x_{i})-H(x),$$

where H(x)=−∫p(x)logp(x)dx designates the information entropy. MI measures any correlation in the system, both linear and higher order. FCA minimizes the MI between the collective coordinates in the protein system, of which the coordinates from the PCA are used as an initial estimate. The FCA modes were subsequently ordered by anharmonicity rather than amplitude and the analyses presented represent the modes with the highest anharmonicity. For all of the protein–ligand systems studied in this work, the top two most anharmonic modes described ≥82% of the fluctuation of the entire system and therefore are used to describe the functionally relevant motions of the protein–ligand systems.

##### Determining Allosteric Network Pathways in c-Type Lysozymes.

Multiple Sequence Alignment

The MSA data set was retrieved from the Pfam database (Punta et al. 2011) (PF00062). A PDB reference sequence was chosen and iteratively aligned with each sequence in the MSA using the Smith–Waterman algorithm (Smith and Waterman 1981). Each matched sequence was included if it had at least 95% sequence identity with the reference PDB. Based on the residue mapping between the PDB sequence and the matched sequences, the columns of the MSA were truncated so as to retain those residues structurally resolved in the PDB sequence. From the truncated set of sequences, redundant sequences in the MSA were removed by using a threshold of 99% to eliminate the sequences that had more than 20% gaps. In the end, the refined MSA contained 2,026 sequences in 415 clusters (Sievers et al. 2011) with a minimum sequence identity of 53%. The reference sequence and structure were set as HEWL with the PDB code 1lyz.

##### Conservation and Coevolution Analysis.

The conservation and coevolutionary analyses on the c-type lysozyme family sequences were carried out with the MISTIC (Simonetti et al. 2013) server. The MI score as implemented in MISTIC is calculated between pairs of columns in the MSA. The frequency for each amino acid pair is calculated using sequence weighting along with low count corrections and compared with the expected frequency. It is assumed that mutations between amino acids are uncorrelated. The MI score is calculated as a weighted sum of the log ratios between the observed and expected amino acid pair frequencies. The coevolution propensity is evaluated using the method of Dunn et al. (2008) to minimize the background MI signal for each pair of residues and the effects of shared ancestry in evaluating coevolutionary patterns. The MI scores were translated into MI *z*-scores by comparing the MI values for each pair of positions with a distribution of prediction scores obtained from a large set of randomized MSAs (Buslje et al. 2009). The *z*-score is then calculated as the number of standard deviations that the observed MI value falls above the mean value obtained from the randomized MSAs. A *z*-score threshold of 6.5 describes a sensitivity of 0.4 and a specificity of 0.95. MISTIC lists every MI value between two residues with a value ≥6.5.

##### Visualization of Networks.

The positions of the nodes in the networks were determined with a two-step process of classical scaling and stress minimization (Brandes and Pich 2009) using the software tool visone (Baur et al. 2002). Network communities were calculated with modularity maximization (Newman 2006) using the software tool ORA (Carley et al. 2012).

## Supplementary Material

Supplementary material S1–S7 are available at *Molecular Biology and Evolution* online (http://www.mbe.oxfordjournals.org/). 

Supplementary Data
