# Supplementary material for: Using THz Spectroscopy, Evolutionary Network Analysis Methods, and MD Simulation to Map the Evolution of Allosteric Communication Pathways in c-Type Lysozymes
Source: Mol Biol Evol. 2015 Sep 3;33(1):40–61. doi: 10.1093/molbev/msv178 (PMC4693973; doi:10.1093/molbev/msv178)
Supplement: Supplementary Data [file supp_33_1_40__index.html]

Using THz spectroscopy, evolutionary network analysis methods, and MD simulation to map the evolution of allosteric communication pathways in c-type lysozymes — Using THz Spectroscopy, Evolutionary Network Analysis Methods, and MD Simulation to Map the Evolution of Allosteric Communication Pathways in c-Type Lysozymes — Using THz Spectroscopy, Evolutionary Network Analysis Methods, and MD Simulation to Map the Evolution of Allosteric Communication Pathways in c-Type Lysozymes — Using THz Spectroscopy, Evolutionary Network Analysis Methods, and MD Simulation to Map the Evolution of Allosteric Communication Pathways in c-Type Lysozymes — Supplementary Data 

# Using THz Spectroscopy, Evolutionary Network Analysis Methods, and MD Simulation to Map the Evolution of Allosteric Communication Pathways in c-Type Lysozymes

## Supplementary Data

files

- Supplementary Data - pdf file
