# Supplementary material for: Using THz Spectroscopy, Evolutionary Network Analysis Methods, and MD Simulation to Map the Evolution of Allosteric Communication Pathways in c-Type Lysozymes
Source: Mol Biol Evol. 2015 Sep 3;33(1):40–61. doi: 10.1093/molbev/msv178 (PMC4693973; doi:10.1093/molbev/msv178)
Supplement: Supplementary Data [file supp_msv178_Supplementary.pdf]

S1.Energy landscape theory of protein structure and dynamics and the formation of allosteric communication pathways

Internal protein motions take place on a diverse range of time scales. It has been proposed that the range of dynamical motions in a protein are arranged in a hierarchical tier of interrelated conformations that reside within closely spaced energy levels. In this representation, protein motions are heterogeneous and those sharing similar energies within a particular conformation are said to exist as substates within the possibility of protein configurations. Each of these substates reflects a local minimum on the protein free energy landscape and corresponds to a specific protein structure that is accessed via thermal fluctuation. Large-scale conformational changes in proteins require moving from one protein conformational state to another and typically occur on relatively long time scales (nanoseconds to milliseconds), whereas the fast fluctuations within or between a specific substate happen on a much shorter time scale (femtoseconds to picoseconds) and function as preliminary steps that guide the longer time scale conformational changes associated with protein function (Fig. S1).

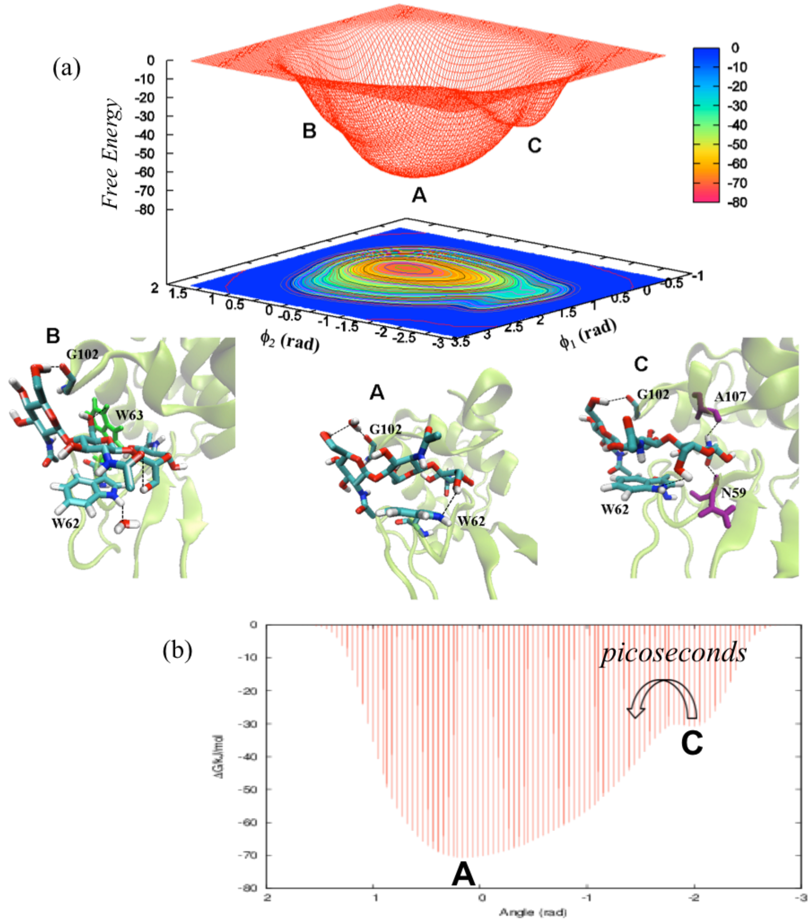

Figure S1: (a) Free energy surface (FES) representation of the twisting motion of the rings on subsites A and C of $(NAG)_3$ bound to lysozyme. The three main minima in the FES (labeled as A, B, and C) reflect conformational substates[1] of lysozyme – $(NAG)_3$. (b) 2-D representation of the FES from (a) depicting the movement from one protein conformational substate (substate C) to another (substate A) via thermal fluctuation on the picosecond time-scale.

Allosteric regulation enables the activity of one site in a protein to modulate function at another spatially distinct region. Frequently allosteric communication in proteins represents informational flow between functional surfaces of signaling proteins and/or regulation of

properties through molecular interaction. Further, allosteric regulation is mediated by the interplay between long-range interactions linked with large-scale protein structural changes and fast, internal fluctuations associated with entropy-driven conformational changes. It is interesting to note that the commonly accepted interpretation of allostery is based on the ensemble view of pre-existing conformational states. In this interpretation, pre-existing pathways can be modified by dynamic perturbations that shift the relative distributions of ensembles resulting in new or altered protein functions. Interestingly, it has also somewhat recently been determined from computational and to some extent experimental sequence evolution analyses that allostery emerges in the evolution[2] of a protein resulting in a range of different mechanisms that can be observed when contrasting individual members of a single protein family.

S2. Calmodulin and torsional oscillations that promote long-range coherence in the protein structure

Calmodulin (CaM) is a calcium binding intermediate messenger protein that is expressed in all eukaryotic cells. It transduces calcium signals by binding calcium ions (Ca^{2+}) and then modifying its interactions with various target proteins. Additionally, both experimental and computational investigations on calmodulin have shown that side-chain torsional motions rather than large-scale backbone motion are linked with propagating signals over long-length scales in the structure of the protein. As part of this investigation, we find that the low-frequency THz spectra of apo- and holo- CaM in Fig. S2 have similar peak positions suggesting that the structure of the protein does not change appreciably when bound with calcium. However, there are clear differences in the intensity of the detected modes in the liganded protein when contrasted with the un-liganded state indicating that the amplitude of motions is notably altered in the bound state. In both cases, the dominant peak in the global mode spectrum is located at approximately 20 cm^{-1} and is ascribed to non-polar side-chain torsional fluctuations. The dominance of this mode in the low-frequency spectra is likely related to internal fluctuations that are central to CaM function. It is known that cooperative side-chain fluctuations in CaM are an essential characteristic of its function and are tied with fine-tuning recognition and optimizing binding to target proteins. Interestingly, there are several other unresolved peaks in the experimental spectra that can be traced to other types of collective protein motions such as backbone and mainchain fluctuations that appear to be less dominant in CaM dynamics at functional temperatures. It is probable that the lack of defined features in the experimental spectra (Fig. S2) is due to the small size of the protein combined with the considerable motional freedom in CaM that develops as a result of correlated side-chain fluctuations, that coincidentally, also have important implications with respect to cellular signaling[3,4].

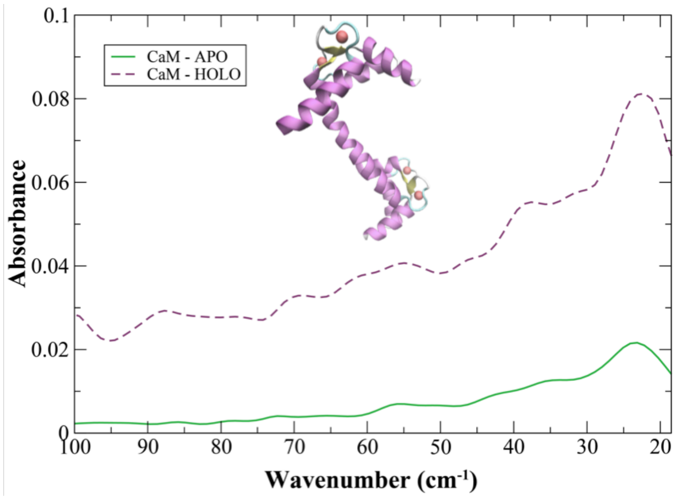

Figure S2: Experimental THz spectrum of hydrated, unoriented film samples of apo-CaM (green, solid line) and holo-CaM (purple, dashed line) in the 20 – 100 cm^{-1} spectral region at 298 K. Inset shows a cartoon representation of CaM bound with calcium ions.

S3. HAMLET and microscopic protein-ligand interactions involved with cytotoxic activity

Utilizing MD simulation as a means of probing the most prominent features of the conformational fluctuations that develop in response to OA binding, we identify key residues that may form the foundation of the targeted specificity in HAMLET cytotoxic activities. For instance, the network view of the residues that are

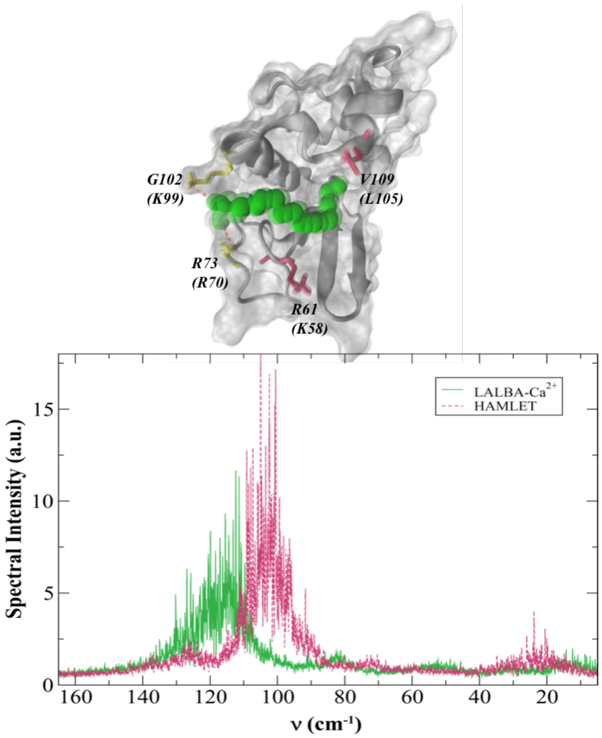

Figure S3: (Top) Cartoon representation of the HAMLET complex overlaid with a space-filling representation illustrating the interaction of OA with residues in the active-site cleft from the MD simulation. The numbering of the residues and the residue itself is based on the reference structure 1LYZ.pdb used in the MSA, while the residue number and residue in parentheses reflects the numbering from the LALBA structure used in the MD simulation. (Bottom) Fourier Transform of the VACF of Val 109 (L105) from the MD simulation of LALBA-Ca^{2+} (green, solid line) and HAMLET (red, dashed line) at 300 K.

stimulated in response to ligand binding reveals that the ligand motion and protein collective dynamics are intimately connected in HAMLET. In the HAMLET complexes investigated, the OA molecule has a preferential binding affinity in the protein's catalytic binding cleft. In this orientation, Arg 61, which is located at the edge of the active-site cleft, couples with the dynamics of OA by means of hydrophobic interaction (Fig. S3). This coupled ligand-amino acid fluctuation affects both the local and the global dynamics of the protein and propagates outward from the binding site towards surface residues that regulate both the stability and positioning of the flexible loop that forms the interaction site that mediates contact with other macromolecules. Namely, the collective, torsional fluctuation caused by the protein-ligand hydrophobic interaction extends to the protein exterior causing rotational flexibility in the region consisting of Val 109 and Ala 110 on Helix D and Phe 34 on Helix B. The conformational dynamics of the residues excited on the surface of the protein through protein-ligand coupling have been shown to have a strong effect on the binding of other functional groups to the protein exterior. Hence, the protein-ligand coupling may play an essential role in the evolution of the specificity and recognition properties of HAMLET that set it apart from EL-OA in terms of targeted tumoricidal activity. Arg 61 and Val 109 are both residues with intermediate sequence entropy and they are also highly central within their distinctive network communities (Fig. 3). Val 109 has hydrophobic side-chains that are exposed to the protein exterior. Ligand binding of OA to LALBA increases the overall intermolecular hydrophobic interaction sites on the protein surface by activating strong links to Val 109 as well as to select amino acid residues within the same community as Val 109. The newly acquired link formed by the connection of Val 109 with Arg 61 in HAMLET not only transmits the torsional dynamics of the ligand directly to specific regions on the protein surface, but also appears to be connected with an adapted mechanism for orchestrating the dynamics associated with orientation at the protein surface for regulating macromolecular recognition (Fig. S3).

From MD simulations that we have conducted on HAMLET we have uncovered that when the temperature of the complex is lowered below the transition temperature (T_g) it leads to an enhancement of hydrophobic group hydration on the HAMLET surface. This enhancement in the hydration features of the protein-oleic acid complex is attributed to more favorable water interactions at the solvent-protein interface. Even more interesting is that the solvent-induced packing defects that develop on the protein surface at the lower temperatures have a direct influence on protein structural stability and consequently are also interrelated with the amplitude

of protein internal, structural fluctuations. The surface defects that arise from the temperature-dependent solvent interactions propagate to the protein interior via increased structural fluctuations on the protein surface that are transmitted to the protein interior as packing defects that have a destabilizing effect on the overall global, protein structure. The packing perturbations in the hydrophobic core region that accompany solvent reorganization on the protein surface can be observed experimentally as changes in the global dynamics of the protein as a function of temperature. For instance, in Fig. 1d we find that the HAMLET complex has prominent peaks in the 25 – 40 cm^{-1} spectral region below T_g, but these peaks decrease in distinction and amplitude in the experimental spectra as the temperature in increased. The peaks in the 25 – 40 cm^{-1} spectral region reflect global (large area) surface fluctuations involving primarily protein, (polar and exposed non-polar) side-chain motions. The decrease in prominence of these spectral bands as the temperature is increased reflects a reduction in packing deformations in the protein core and a consequent increase in the global tertiary structure. For instance, in Fig. 1d, not only do we notice a decrease in the bands associated with solvent-induced side-chain fluctuations when the sample temperature is increased above T_g, but we also observe an increase in the spectral signature associated with correlated backbone dynamics at 80 cm^{-1} that is a result of a more stable protein tertiary structure. Also of interest is that experimentally, we also detect that the local distribution of water molecules on the protein surface is altered with the increase in temperature in the 100 – 200 cm^{-1}spectral region (not shown here but will be elaborated on in a future publication). Moreover, the localized protein-solvent contact variations detected also seem to be distinctly tied with modifications in protein structural stability.

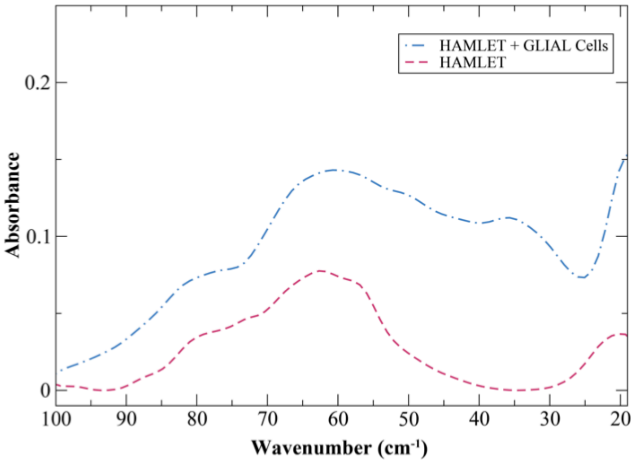

Figure S4: Experimental THz spectrum of HAMLET (magenta, dashed line) and HAMLET in the presence of human glioblastoma cells (blue, dotted-dashed line) at 300 K.

One interesting aspect of experiments conducted on HAMLET is that we observe a similar trend in low-frequency dynamics when the protein is in the presence of tumor cells (at room temperature) as when the temperature of the protein complex is lowered below T_g (Fig. S4). Specifically, the tumoricidal active complex of HAMLET uncovers the formation of a new peak in the region associated with protein surface fluctuations centered at about 35 cm^{-1} at room temperature and this is accompanied by an increase in peak amplitude of all other observable peaks in the low-frequency region. The development of the new peak at approximately 35 cm^{-1} in the global dynamics of the HAMLET-tumor cell complex is complemented by detectable modifications in the hydration shell dynamics of solvent molecules surrounding specific regions on the protein surface in the higher frequency region of the THz spectrum as well as variations in intra-protein interactions in the protein hydrophobic core region that direct the dynamic stability of the protein tertiary structure (not shown). Together these preliminary results suggest that there is a connection between the excitation of HAMLET surface interactions that occur during contact with cancerous cells that are fundamentally intertwined with stability changes in the protein core that may be important for developing a molecular level understanding of how HAMLET (i) is recognized and/or internalized by tumor cells while healthy, undifferentiated cells are less affected and (ii) once internalized, activates molecular interactions that ultimately initiate cell death.

S4. Experimental detection of global communication networks in c-type lysozymes and the relationship to inter-domain coupling

THz spectroscopy of global protein fluctuations not only provides insight into the sampling of conformational substates but also affords direct information about specific protein intra- and intermolecular interactions that are characteristically tied with the chemistry of energy barrier transitions in enzymatic reactions. For instance, we have determined that the 60 cm^{-1} experimental mode in Fig. 6 represents induced, intra-protein H-bond fluctuations. Correspondingly, the 80 cm^{-1} experimental band reflects an in-plane collective backbone torsion that emerges in the protein samples as a result of weakening of intramolecular interactions within the protein core as well as coupling to (localized) solvent relaxation modes that are activated in response to the hinge bending motion that develops within the group of homologous proteins. Together, the induced mechanisms promote cooperative motion between the two separate domains in the representative protein structures that ultimately fosters inter-domain coupling. In Fig. 6 we note that lysozyme-$(NAG)_3$ and calcium bound LALBA have prominent peaks at *both* 60 cm^{-1} and 80 cm^{-1} in the experimental spectra suggesting that the dynamics of the hinge region have a profound effect in the global network of interactions in the representative proteins. Analogously, we find that the experimental peaks are less conspicuous in both the HAMLET and EL-Ca^{2+} experimental spectra, which is in line with our computational measurements and indicates that the dynamics associated with inter-domain coupling is reduced in both samples. Generally, hinge-bending motion involves changes in protein mainchain dynamics that is accompanied by packing interaction modifications near the domain barrier. In the studied protein structures, we find that the network of densely organized molecular interactions in the vicinity of the hinge-bending site mediate allosteric regulation. The presence of a collection of stable, intramolecular interactions near the binding pocket of lysozyme-$(NAG)_3$ and LALBA ensure structural stability of the hinge site; however, intermolecular contact variations in the same region facilitate cooperative movements between the domains by modifying the dynamic ensemble of conformational substates. We have previously noted that a lack of inter-domain coupling is connected with stronger (hydrophobic) core protein interactions and in turn, greater localized protein flexibility that can be directly detected in the experimental higher frequency THz region (Fig. 4).

S5. Betweenness centrality and the importance of Asn 19 in the evolutionary network

i. Betweenness Centrality

Betweenness centrality C^B for a node n counts the number of times the node n is on shortest paths between all other pairs of nodes [5]:

$$C^B(n) = \sum_{i<j} \frac{g_{ij}(n)}{g_{ij}}$$

With g_{ij} as the number of shortest paths between i and j and $g_{ij}(n)$ the number of shortest paths between i and j of which n is a part.

The central underlying assumption of this metrics is that there exists some form of activity/energy in the network that flows through the entire network *only* on shortest paths.

Based on this definition, nodes are central when they lie on many shortest paths between other pairs of nodes. This is the case for nodes that connect different areas of the networks, i.e. bottlenecks, bridges. For instance, Asn 19 has high betweenness centrality in our networks because it constitutes the shortest connection between the communities labeled with blue and red nodes. Similarly, Thr 40 and Trp 111 receive high betweenness centrality from building bridges between the blue and the yellow community. In contrast, Val 109 gets its centrality from being a hub in a less densely connected part of the network.

ii. The effect of removing Asn 19 and Tyr 20 from the MSA network

If Asn 19 and Tyr 20 are removed from the network, we notice that the network structure is dramatically altered. For instance, it becomes apparent that the blue/red community connection is removed. Consequently, betweenness centrality is accumulated at residues Thr 40 and Trp 111. Betweenness centrality of these nodes, as well as their neighbors (e.g. His 15 and Cys 94), is increased. Interestingly, Val 109 is still in a top position of the ranking indicating that the centrality of this residue is not a result from the flow between different communities but from the central position within the red community.

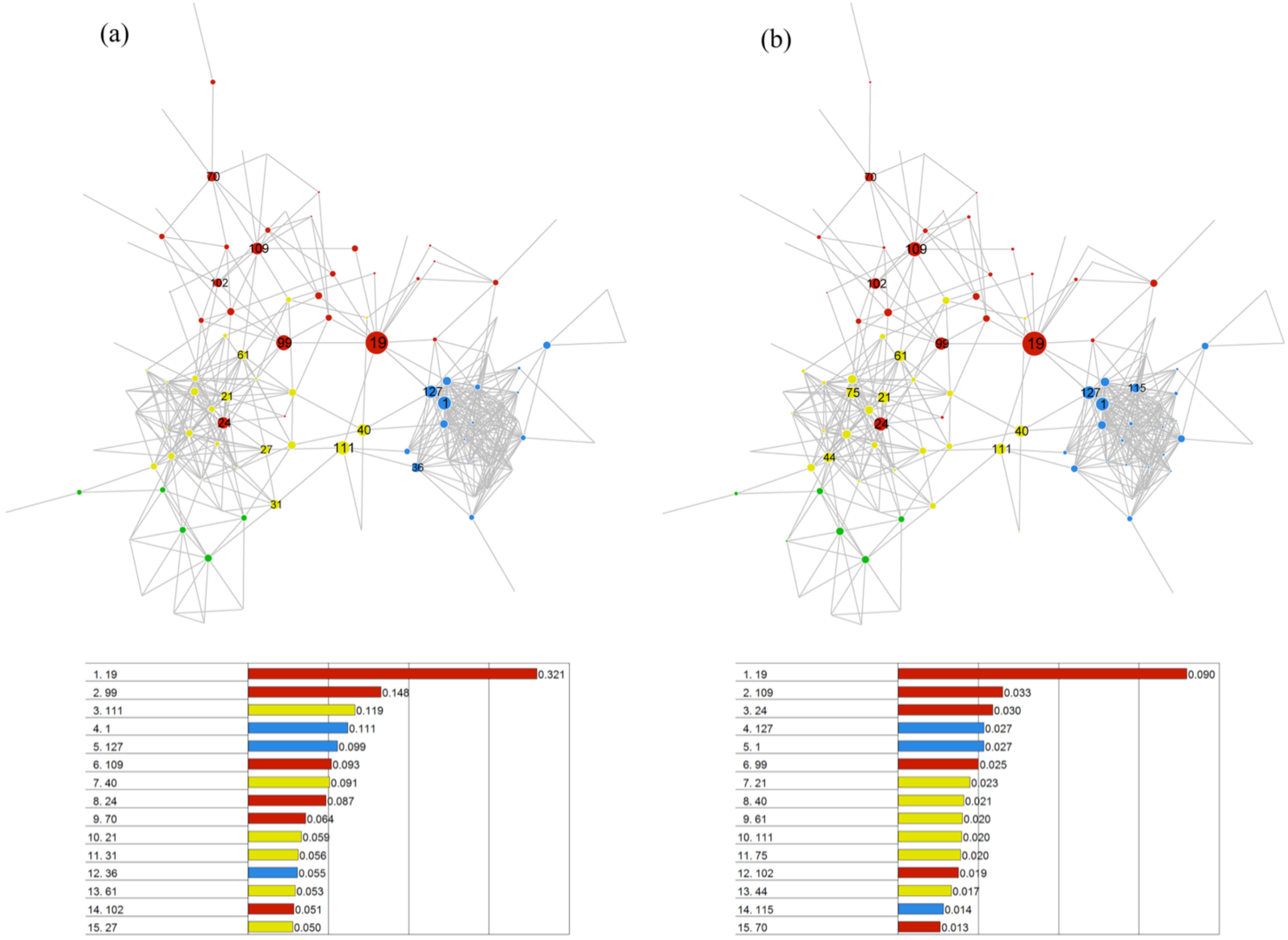

Figure S5: Betweenness centrality in the sequence evolutionary network. Larger nodes represent residues that are more often on shortest paths between other pairs of residues. The top 15 residues are labeled. (a) Regular betweenness centrality based on all shortest paths in network. (b) k3-betweenness centrality disregarding paths with distance longer than three.

iii. k_3-betweenness centrality

One way to modify betweenness centrality is to restrict the path length of the shortest path calculations. In other words, instead of considering shortest paths between all pairs of nodes, one just considers only paths with length of *k* or shorter [6]. We apply this metric with k=3. With this approach global bridges become less significant and in its place, well-connected residues in

different parts of the network lead the ranking. Intriguingly, Asn 19 scores again highest, as this residue is not just a global bridge but also has a large number of connections.

S6. Solvent influence in allosterically shaping protein conformational landscape in c-type lysozymes

One of the more interesting resonances observed in the experimental spectra of the c-type lysozyme protein structures studied is the ~140 cm^{-1} mode that is featured prominently in the apo-proteins LYZ[7,1] and MG-LALBA, but is also visible in both the EL-Ca^{2+} and HAMLET spectra obtained in this study in Fig. S6. In general, solvent-protein picosecond time scale coupled dynamics seems to play an important role in allosterically influencing the conformational energy landscape of proteins in the family. For instance, in a previous investigation[1] it was determined that the ~140 cm^{-1} peak in lysozyme is attributed to a translational mode involving solvent molecules in a structured (tetrahedral) configuration that interact with rigid, exposed helical regions in the protein secondary structure. The solvent-protein interactions create a heterogeneous conformational space in the protein energy landscape by coupling protein motions to fast (picosecond time scale) H-bonding solvent fluctuations. The end result is a “smoothed-out” energy surface with distinct ensembles separated by low energy barriers that enable the protein to sample the substates that map out the enzyme conformational landscape. On the other hand, the MG state of LALBA coexists in an ensemble of multiple subsets of hydrophobic clusters[8] that act to stabilize the partially folded protein intermediate state. This dynamic ensemble of partially-folded side-chain clusters are highly hydrated and the fast protein-solvent H-bonding interactions lead to a disparate collection of exposed protein regions with differing extents of intra-protein packing distortions and areas of local mobility. The 140 cm^{-1} mode in MG-LALBA reflects the hydration of the more stable helical regions in the protein structure, while another peak at 130 cm^{-1} relates to protein mainchain – solvent H-bonding torsional fluctuations of water molecules that interact with localized, flexible regions on the protein surface. In both cases, we detect that the non-liganded systems have considerable conformational heterogeneity when compared with their ligand-bound protein counterparts. Furthermore, the role of the solvent appears to be decisive in defining the protein conformational space.

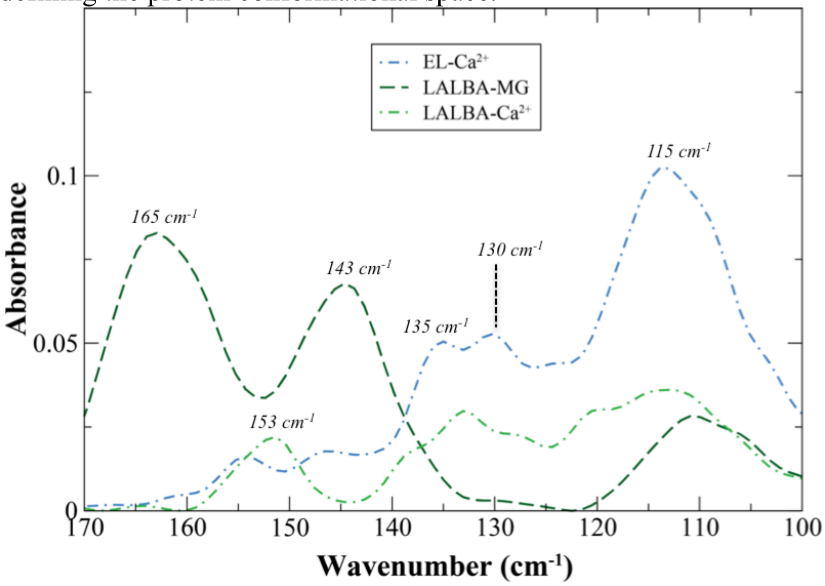

Figure S6: Experimental THz spectrum of EL-Ca^{2+} (blue, dotted-dashed-dotted line), LALBA-MG (dark green dashed line), and LALBA-Ca^{2+} (light green, dotted-dashed-dotted line) in the 100 – 170 cm^{-1} spectral region at 300 K.

S7. Force distribution analysis and networks of evolutionarily coupled residues in the c-type lysozyme subfamily

In this investigation we have also extended the MD simulations of protein structures in the c-type lysozyme family to include force distribution analyses (FDA)[9,10]. The aim of these studies is to deduce the mechanical strain that is both distributed and propagated within the interior of the protein due to the protein-ligand interactions that take place within the protein ligand-binding site and/or active-site. It has already been mentioned that biomolecules have been conjectured to exist in a range of closely related conformational states (or substates) and this is referred to as an ensemble. Incidentally, allostery is a direct result of this ensemble. A perturbation, such as ligand-binding, in the protein structure leads to a shift in the distribution of conformational states across the ensemble[11]. And a return to equilibrium requires a release of that strain energy through a series of propagating structural deformations. So interestingly, allosteric signal propagation in proteins is mostly accompanied by small, structural rearrangements rather than large-scale protein conformational changes. Therefore, to comprehend the nature of the network of correlated protein fluctuations that arise in response to ligand-binding in the c-type lysozyme protein sequences (and consequently, also form the basis of allosteric communication in the protein family) we have conducted PCA on the residue averaged pair-wise forces in the representative protein structures when bound to their characteristic ligands. Hence, in this work, the interpretation of the propagation pathways uncovered from FDA relies on the observations from force-PCA analyses.

i. Equine lysozyme bound with calcium. From the MD simulations, we are able to establish a network of interactions that propagate internal strain from the ligand-binding site to the rest of the protein via subtle, structural fluctuations. In EL-Ca^{2+} the force from binding is almost exclusively concentrated on residues in contact with the calcium-ion ligand (Fig. S7a). Specifically the force is focused mainly on the three binding residues, Ser 85, Ala 90, and Ser 91 (the binding aspartates, Asp 85, Asp 90, and Asp 91 in EL). The excess energy from binding is transmitted through the protein structure in a localized manner via small-scale, rotational oscillations that invoke a network of spatially connected residues in the hydrophobic core that are, for the most part, also conserved. Specifically, the torsional motion of Leu 83 in the calcium-binding pocket propagates the strain energy away from the ligand-binding site into the protein core by correlated (in-phase) torsional fluctuations of side-chains involving conserved residues in the β-hairpin loop and a limited portion of the active-site binding pocket. The residues involved include Gly 54, Ile 55, and Gln 57 as well as the active-site residue Ser 36. Similarly, out-of-phase collective oscillations connect the dynamics of the hairpin structure with coevolved residues in the outer portion of the active-site cleft. In this case, the residues activated during the signal propagation process mostly involve coevolved protein core residues Gly 104 and Trp 108 that form regions of the (mobile) outer segment of the active-site binding pocket and the hydrophobic box region, respectively. Principally, the high mutual information (MI) residues comprising the flexible region of the active-site cleft (Val 99 – Gly 104) couple with slight adjustments in the hydrophobic box region to actively spread the excess energy to interfacial residues on the protein surface. The mechanism of propagation utilizes out-of-phase correlated oscillations of the two combined regions of the protein (residues lining the protein active-site and residues residing in the hydrophobic box) to establish long-range communication that directly connects flexible loop residues (residues 67 through 69) in the long-loop region in the β-domain with residues that form the A/B loop and a small portion of the 3_{10}- helix in the α-domain. The culmination of excitation from ligand-binding ultimately results in a highly, mobile region of localized flexibility on the protein surface centered about the A/B loop and also including a very narrow portion of the 3_{10}-helix in the α-domain. It is also worth noting that the allosteric signal propagation process induced by ligand-binding in EL incorporates two distinct mechanisms of excess energy dissipation: short-range interactions initiated by (in-phase) correlated fluctuations of close-proximity, conserved residues within the protein-core and longer-distance connections prompted

by out-of-phase correlated fluctuations between coevolved residue pairs in more exposed regions on the protein exterior.

ii. Equine lysozyme bound with oleic acid (EL-OA)

The force-induced fluctuations in EL-OA (Fig. S7b) are not significantly altered when EL is bound with oleic acid when compared with that of the native state. However, the mechanism of signal transmission from ligand binding differs significantly from that of EL-Ca^{2+}. The OA ligand binds at the edge of the long-loop region of EL, which interestingly, greatly increases the fluctuational amplitude of residues found within the β-sheet. In particular, due to OA binding, the torsional fluctuation of high MI residue Arg 45 is greatly enhanced when compared with other residues in the β-sheet. The overall increased torsional dynamics of β-sheet residues due to perturbation of ligand binding is transmitted through the protein interior via coupled out-of-phase fluctuations with residues encompassing the hydrophobic box region of the protein (Leu 17, Gly 22 – Ser 24, Val 99, and Met 105) and culminate as large-scale fluctuations on the protein surface that mainly involve residues Asn 19, Tyr 20, and to a lesser extent Lys 116. It is interesting to point out that that the dynamics of coevolved protein core residues such as Gly 104 and Trp 108 have motion that is greatly reduced in EL-OA when contrasted with the native state of the protein.

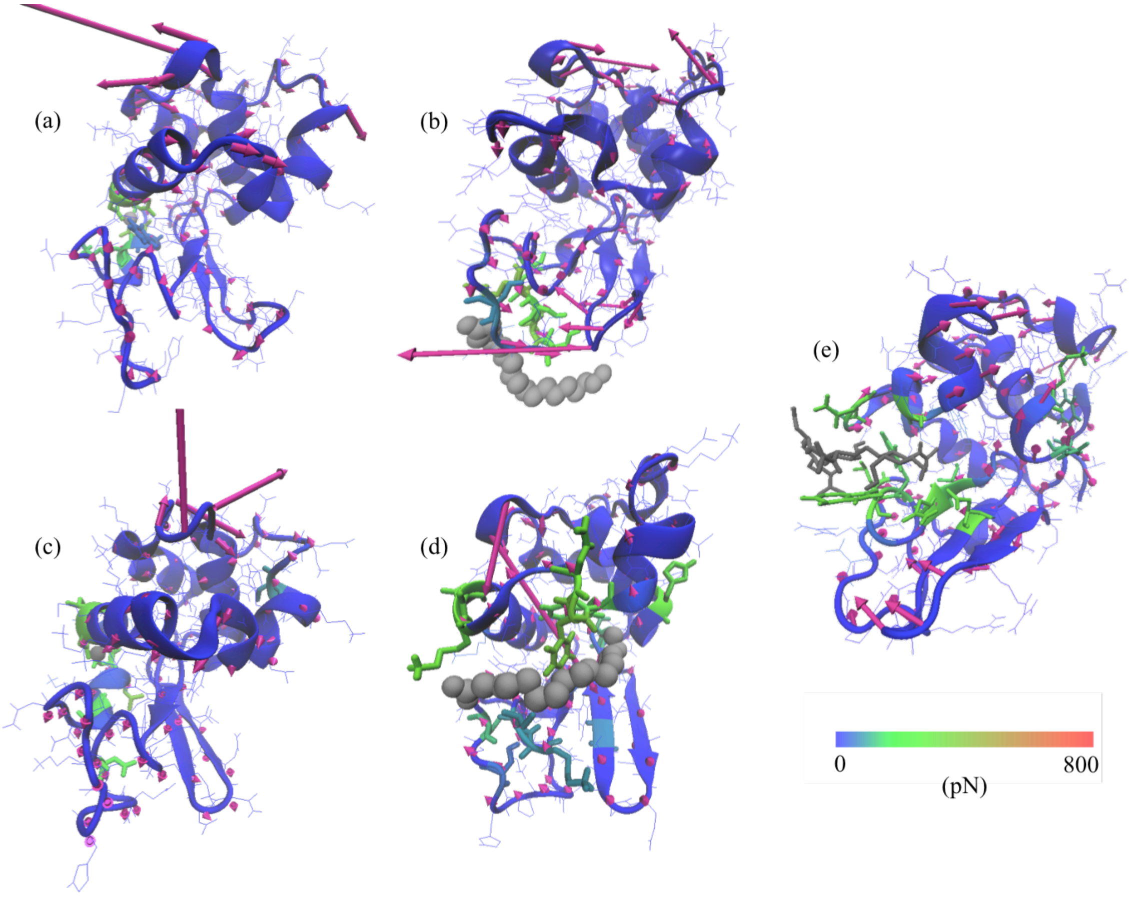

Figure S7: Changes in residue forces (ΔF) mapped onto a cartoon representation of (a) EL-Ca^{2+}, (b) EL-OA, (c) LALBA-Ca^{2+}, (d) HAMLET, and (e) LYZ-$(NAG)_3$. The color scheme ranges from blue to red, where blue describes regions with low changes in residue forces and red high. The residues with the greatest force are shown with a licorice representation. Arrows illustrating the motion from the force-PCA are also overlaid on the cartoon representation of the reference structures.

iii. LALBA bound with calcium. The characteristics of the force-induced fluctuations in LALBA-Ca^{2+} are quite distinct from those observed in EL-Ca^{2+}. Analogous to what has been observed in EL bound with calcium, there is quite a bit of force close to the residues actively involved with binding the calcium ion (Fig. S7c). But unlike, EL the force from ligand-binding is additionally distributed more widely throughout the protein structure. We speculated in earlier sections that the key to long-range communication in LALBA is due to community overlap of coevolved residue pairs. The force distribution analysis makes the mechanism of allosteric communication in the protein easier to interpret. Particularly, the force on Leu 84 from the calcium-bound ligand leads to large-scale translational motion of residues surrounding the ligand-binding site. The large-amplitude motion of the calcium-binding residues is subsequently propagated throughout the protein hydrophobic core by means of correlated, torsional fluctuations connecting calcium-binding residues and hinge-bending residues. Further, the intramolecular interactions and structural modifications that take place around the domain interface in LALBA, that accompany calcium-binding, further facilitate signal propagation throughout the protein 3-D structure by promoting an extensive network of collective oscillations. Particularly, adjustments in the H-bond network of residues surrounding the interface separating the α- and β– domain (or the hinge-bending site) in conjunction with adaptations in the geometry of the binding cleft in the vicinity of Tyr 53 are central for enhancing signal propagation and long-range communication in the protein. Particularly, the large amplitude translational motion of Leu 84 activates correlated oscillations in Tyr 53 that are coupled with hinge-bending residue fluctuations involving Ile 55 and Leu 56 near the intersection of the two domains. Hence, alterations in the arrangement of hydrogen-bonding interactions comprising residues Gln 57, Asn 59, Ala 107, and Trp 108 (Gln 54, Asn 56, Tyr 103, and Trp 104 in LALBA) that are formed in the presence of the calcium ion function to establish a more rigid hydrophobic core, which consequently, also facilitates inter-domain coupling. These collective, large-amplitude rotational oscillations dissipate excess energy that forms as a result of ligand-binding. It is interesting to point out that Tyr 53 is highly conserved in the MSA (Fig. 2a) and is instrumental in forming the pathway of signal propagation in calcium bound LALBA. It is the dynamic coupling between Leu 84 and Tyr 53 that arbitrate cooperative motions between the domains by activating large-amplitude rotational fluctuations near the domain interface. Intriguingly, the rotational oscillations near the hinge-bending site are further progressed through the protein structure by the excitation of packing density structural deformations in the protein core that are also initiated by interaction with Tyr 53. In this case, small-amplitude torsional oscillations excited in the protein hydrophobic core disrupt an optimized network of intra-protein interactions that maintain protein stability. The residues involved include many conserved residues such as residues Ser 36, Asn 59, and Trp 111 and others that are not conserved such as Ala 31 and Phe 34 but are recognized as being important in maintaining the tertiary fold in lysozyme. The small-scale torsional oscillations provide a pathway for the signal to propagate through the protein interior and in doing so also excite long-range correlations that further transmit the excess strain energy out to distant sites in the protein structure. Along these lines, the dynamics of Tyr 53 is also closely associated with the formation of anharmonic dynamics involving residues comprising the β-sheet. Particularly, β-sheet residue Asn 46 has considerable interatomic force and substantial flexibility. Asn 46 is highly conserved in the structural alignment and possesses considerable conformational mobility in the global modes, suggesting that its dynamics may be associated with movement that assists in the mechanism of intra-protein signal transduction by enhancing the long-range connection between the residues in the network. For instance, the motion of Asn 46 (and to some extent the other residues comprising the β-sheet) promotes torsional flexibility in the residues lining the active-site binding cleft. The long-range coupled excitations arise as a direct result of localized, intramolecular contact variations within the protein core that accompany ligand-binding. One of the more significant residues contributing to the anharmonic dynamics surrounding the active-site cleft is high mutual information residue Val 109. The motion of Val 109 is intimately tied with

the dynamics of high sequence variability residues on the protein surface. The surface fluctuations of these adaptable residues have been implicated in the mechanism of creating macromolecular recognition sites on the protein exterior. Principally, prominent fluctuations of residues Gln 121 and Ala 122, which form part of the aromatic I cluster, in addition to amino acid residues Arg 112, Asn 113, and Lys 116 that are recognized antigenic sites in c-type lysozymes. Together the excitation of the exposed residues alter the conformational properties of the protein surface and are likely involved in the process of adjusting the interface for protein-protein contact or recognition.

iv. LALBA bound with oleic acid. When LALBA is bound with oleic acid (OA) to form the HAMLET complex there is a considerable difference in the observed force propagation patterns when juxtaposed with LALBA-Ca^{2+} (Fig. S7d) The oleic acid molecule is bound by weak electrostatic interactions to amino acids Arg 73 and Gly 102 (Arg 70 and Lys 99 in LALBA) in the region surrounding the protein binding-pocket. As a result of binding, there is a strong torque in the active-site cleft that places a large amount of force on residues on the periphery of the binding pocket, namely Lys 61 and Val 99 (Lys 58 and Leu 96 in LALBA). Both Lys 61and Val 99 have high coevolution propensity and low conservation probability indicating that they are involved in mediating substrate specificity. Furthermore, the force distribution pattern reveals that Lys 61 is strongly coupled with the dynamics of the ligand. The protein-ligand coupling modifies the hydrophobic core interactions connected with protein stability and consequently, the alteration in protein tertiary interactions acts as a mediator in long-distance amino acid communication. Similar to what has been observed in LALBA-Ca^{2+}, the force attributed to protein-ligand coupling is transmitted through he hydrophobic core via small-amplitude torsional oscillations. These localized, torsional fluctuations couple with the dynamics of hydrophobic active-site residue Val 109 on the periphery of the binding cleft and subsequently propagate the strain of binding to neighboring residues on the protein surface, principally Gly 102 in the solvent accessible loop bordering the substrate binding cleft as well as Arg 112 and Asn 113 in the exposed helical region of Helix D. The restricted torsional fluctuations resulting from signal propagation are transmitted to the protein surface in a region that has been linked with shaping the protein interface for substrate recognition. On the other hand, the force distribution pattern of Val 99 is diffused more widely in the protein 3-D structure and has a stronger effect on the surface residues in the protein α-domain. Val 99 has a high MI value and is a central feature in the coevolutionary analysis of the MSA. The strain force on Val 99 that emerges from ligand-binding dissipates through weak interactions with other coevolved residues, such as Met 105 and Tyr 23 that form the solvent-accessible edge of the hydrophobic box as well as through the formation of anharmonic dynamics that modify the conformational environment around the substrate binding pocket. The resultant perturbation in the hydrophobic box region is central for fine-tuning the affinity for ligand-binding; and to this end, leads to prominent surface excitations of residues encompassing the A/B loop. The new connections that are activated by the release of the strain energy in the hydrophobic box also produce solvent-mediated fluctuations in the protein-ligand complex that may further refine substrate-binding affinity, while at the same time increasing the interfacial region associated with macromolecular recognition. The solvent-induced fluctuations mainly engage a network of coevolved residues residing in the flexible, surface loop region in the α-domain, comprising residues 101 – 106. Other coevolved residues that experience weak induced correlated fluctuations as a result of the internal strain propagation emanating from the hydrophobic box region include Trp 62 and Asn 74, which form part of the active-site loop and long-loop in the β-domain, respectively. The delimited dynamic coupling between hydrophobic box residues and the exposed, flexible loops in the β-domain further link the dynamics of the ligand directly with dynamics of the protein. Interestingly, all of the residues that experience weak, force-induced ligand interactions are in close proximity and have high coevolution properties. They also appear to serve a dual role in the function of the protein-ligand

complex: (i) positioning the OA ligand into protein active-site and (ii) actively shaping the surface properties of the protein through fluctuation-driven interactions that are transmitted to remote regions on the protein exterior, presumably in an effort to prepare the complex for additional macromolecular interactions. In any case, the excitations that develop in HAMLET in response to the distribution of strain prompted by OA binding culminate to form a large interaction binding-site on the protein surface in the α-domain. Intriguingly, unlike LALBA-Ca^{2+}, there is limited intersection between core residue fluctuations and large-amplitude dynamics involving β-domain residues. Hence, the β-domain of the protein plays a very limited role in the observed functional dynamics of the HAMLET complex. On the other hand, the interaction with OA produces a strain-propagation pattern that shares many similarities to EL-Ca^{2+}. In both protein-ligand systems (El-Ca^{2+} and HAMLET), correlated fluctuations in the hydrophobic box region actively propagate excess energy to interfacial residues on the protein surface. But when contrasted with EL-Ca^{2+}, it becomes apparent that HAMLET contains more intricate intra-protein interactions that possibly lead to more refined ligand-binding interactions and consequently, binding affinity related to its surface properties.

v. Lysozyme bound with the inhibitor (NAG)$_3$. The force distribution pattern of conventional lysozyme (lysozyme) bound with the inhibitor (NAG)$_3$ shares many of the same properties as the protein-ligand systems that we have previously encountered in this study. Although, one striking difference that seems to set lysozyme apart from the other proteins that we have explored is that the residues subjected to prominent forces in the lysozyme-ligand system are mainly involved with optimizing the fit of the ligand into active-site cleft (Fig. S7d). In this context, it also becomes evident that there is an obvious shift away from the importance of hydrophobic box residue fluctuations in the signal propagation process. For instance, some of the residues that bear the greatest force from ligand-induced strain propagation are situated close to the substrate binding-pocket and share dynamical H-bonds with the ligand. They include coevolved binding-site residues Asp 52, Asn 59, Trp 62, and Leu 75 that line the active-site cleft in the β-domain. These residues not only have high MI values but also are arranged in close proximity (in the 3-D structure of the protein) in the β-domain and function to augment the position of the ligand in the active-site cleft. Similarly, residues with moderately high MI values that are located on the surface of the protein in the α-domain, which include Lys 33, Phe 34, Asn 37, and partially buried residue Trp 111, contribute indirectly to the binding properties of the ligand by activating surface fluctuations that modulate the "fit" of the ligand into the binding cleft. More specifically, the fast, correlated fluctuations of the coevolved surface residues create a conduit of instability that couple with the dynamics of the ligand in the protein active-site to facilitate binding. Moreover, the location of the coevolved surface residues corresponds to sugar binding subsites E and F in lysozyme (which are unbound with the inhibitor (NAG)$_3$) and also coincides with the position of residues that form the aromatic cluster I in LALBA. Both the sugar-binding sites in lysozyme and the aromatic cluster of residues in LALBA are known for optimizing binding interactions of substrates in the enzymes. It is interesting to point out that the residues with the highest force values are located at the intersection of two adjoining networks. In one branch the picosecond time scale aromatic ring torsions of Trp 62 direct the correlated fluctuations of neighboring, coevolved residues that assist in situating the ligand into the active-site cleft. The intra-protein H-bonds of the positioning residues produce a rigid network of interactions that efficiently transmit the changes of force from the active-site that accompany ligand binding out to distant regions in the protein structure. We have previously conjectured that the intra-protein H-bonds involved in positioning the ligand into the binding cleft in lysozyme lead to a decrease in conformational entropy that accordingly, also forms the basis of enzyme specificity. This supposition is further supported by considering the outcome of the two intersecting network arms that are fostered by the intra- and inter-protein forces that are invoked upon ligand-binding. For instance, the other network branch is headed by Gly 102, which serves a pivotal role by coupling

solvent-mediated modes with large-amplitude oscillations of surface residues in the α-domain that effectively regulate the affinity of the ligand. As an aside, it is interesting to point out that the pathway of signal transmission to the protein surface in lysozyme is similar to what has been observed in the HAMLET complex. The solvent-mediated dynamics of Gly 102 couple with oscillations involving hydrophobic active-site residue Val 109 (on the border of the active-site binding cleft) to further communicate the force from ligand-binding out to adjoining residues on the protein surface. The surface residues affected by interaction with Val 109 include Arg 112 in Helix D and Gln 121 – Trp 123 in the 3_{10}- helix of the α-domain. The ligand-induced torsional mobility of these surface residues has been implicated in the mechanism associated with modulating the affinity of the ligand in the substrate-binding cleft. To recap, the Gly 102 side of the network becomes more dynamic by coupling to localized, relaxation modes in the solvent H-bonding network as well as to localized, large-amplitude surface fluctuations in the α-domain. As a result of the coupled dynamics, the increased localized mobility of the loop in which Gly 102 resides, initiates a hinge-bending torsion that unites the correlated fluctuations of coevolved residues in the α-domain with those in the β-domain and in doing so, also links the two distinct network branches. Therefore, the hinge-bending motion of the protein backbone acts as a transducer that enhances the propagation of the dynamic effect of binding out to distant sites in the protein structure. And the convergence of the two, separate network branches plays an important role in moderating ligand specificity. The hinge movement of the domains directs the pathway of force propagation through the protein structure such that it connects the perturbation-induced succession of conformational fluctuations, both adjacent and distant from the substrate-binding site, that function to adjust the shape and geometry of the binding cleft and as an effect, also moderates ligand specificity.

Materials and Methods

i. Calmodulin (CaM) THz spectroscopy experiment

Calmodulin from bovine testes was purchased from Sigma-Aldrich. The samples were initially passed through a desalting column and then resuspended in a double-distilled Tris-HCl, pH 8.0 buffer prior to use. In the apo- protein calcium ions were removed by adding excess EDTA (3.5 mM EDTA), while adding 10 mM $CaCl_2$ to the buffer solution created the calcium-bound sample. Unoriented film samples were prepared for experiment in an analogous manner that has been previously described in the Materials and Methods section of the manuscript.

ii. THz spectroscopy of HAMLET complexed with glioblastoma cells

Human gliobastoma cells (ATTC Cell Lines, Manassas, VA) were prepared by buffer exchange of the cells into a buffer of 10mM NaH_2PO_4 and 0.01 mM EDTA at pH 7.0. The cell suspension was pelleted by centrifugation at 500g and mixed in a 10:1 protein molar ratio (HAMLET:glial cell) with previously prepared samples of HAMLET (preparation detailed in Materials and Methods section of manuscript). Protein films were created from the HAMLET-cancer cell mixture for THz spectroscopy experiments (the preparation of protein films samples used in THz experiments has previously been described in Materials and Methods) and contrasted with HAMLET samples in the absence of glioblastoma cells prepared in the same manner.

iii. *Force Distribution Analysis (FDA)*

A modified version of Gromacs 4.5.3 was used to write out forces, F_{ij} between each atom pair i and j. Forces include contributions from the electrostatic and van der Waals interactions involving the ligand and the protein that are calculated below a certain cut-off distance. These non-bonded pairwise forces of the atom pairs in close proximity comprise a force-propagation network involving short-range to medium-range connections that are averaged over the simulation time[9]. The averaged forces were saved every 10 ps and convergence was reached when an equilibrium value for the forces was attained. The stored forces were written out as force trajectories and the average of those forces were used later for further analysis in R as well as visualization in VMD (http://www.ks.uiuc.edu/Research/vmd/).

References

1. Woods, K. N. THz time scale structural rearrangements and binding modes in lysozyme-ligand interactions. *J. Biol. Phys.* **40,** 121–137 (2014).
2. Süel, G. M., Lockless, S. W., Wall, M. A. & Ranganathan, R. Evolutionarily conserved networks of residues mediate allosteric communication in proteins. *Nat. Struct. Mol. Biol.* **10,** 59–69 (2003).
3. Smith, D. M. A., Straatsma, T. P. & Squier, T. C. Retention of Conformational Entropy upon Calmodulin Binding to Target Peptides Is Driven by Transient Salt Bridges. *Biophys. J.* **103,** 1576–1584 (2012).
4. Lee, A. L., Kinnear, S. A. & Wand, A. J. Redistribution and loss of side chain entropy upon formation of a calmodulin–peptide complex. *Nat. Struct. Mol. Biol.* **7,** 72–77 (2000).
5. Freeman, L. C. A set of measures of centrality based on betweenness. *Sociometry* **40,** 35–41 (1977).
6. Pfeffer, J. & Carley, K. M. k-Centralities: Local Approximations of Global Measures Based on Shortest Paths. in *Proceedings of the 21st International Conference Companion on World Wide Web* 1043–1050 (ACM, 2012). doi:10.1145/2187980.2188239
7. Woods, K. N. Solvent-induced backbone fluctuations and the collective librational dynamics of lysozyme studied by terahertz spectroscopy. *Phys. Rev. E* **81,** 031915 (2010).
8. Mok, K. H., Nagashima, T., Day, I. J., Hore, P. J. & Dobson, C. M. Multiple subsets of side-chain packing in partially folded states of alpha-lactalbumins. *Proc. Natl. Acad. Sci. U. S. A.* **102,** 8899–8904 (2005).
9. Stacklies, W., Seifert, C. & Graeter, F. Implementation of force distribution analysis for molecular dynamics simulations. *BMC Bioinformatics* **12,** 101 (2011).
10. Stacklies, W., Vega, M. C., Wilmanns, M. & Gräter, F. Mechanical Network in Titin Immunoglobulin from Force Distribution Analysis. *PLoS Comput Biol* **5,** e1000306 (2009).
11. Nussinov, R. & Tsai, C.-J. Allostery in Disease and in Drug Discovery. *Cell* **153,** 293–305 (2013).
